# Five nuclear protein-coding markers for establishing a robust phylogenetic framework of niphargid crustaceans (Niphargidae: Amphipoda) and new molecular sequence data

**DOI:** 10.1016/j.dib.2019.104134

**Published:** 2019-06-12

**Authors:** Ajda Moškrič, Rudi Verovnik

**Affiliations:** a Agricultural Institute of Slovenia, Hacquetova ulica 17, SI-1000 Ljubljana, Slovenia; b University of Ljubljana, Biotechnical Faculty, Jamnikarjeva 101, SI-1000 Ljubljana, Slovenia

**Keywords:** Nuclear protein-coding loci, EPRS, Opsin, Arginine kinase, Phosphoenolpyruvate carboxykinase, Glyceraldehyde phosphate dehydrogenase, Phylogenetic analyses, Nucleotide sequences, PCR, Niphargidae

## Abstract

The data presented here includes selection of 5 successfully amplified protein-coding markers for inferring phylogenetic relationships of the family of amphipod crustaceans Niphargidae. These markers have been efficiently amplified from niphargid samples for the first time and present the framework for robust phylogenetic assessment of the family Niphargidae. They are useful for phylogenetic purposes among other amphipod genera as well. In detail, the data consists of two parts: 1. Information regarding markers, specific oligonucleotide primer pairs and conditions for PCR reaction that enables successful amplification of specific nucleotide fragments. Two pairs of novel oligonucleotide primers were constructed which enable partial sequence amplification of two housekeeping genes: arginine kinase (ArgKin) and glyceraldehyde phosphate dehydrogenase (GAPDH), respectively. Additionally, 3 existing combinations of oligonucleotide primer pairs for protein-coding loci for glutamyl-prolyl tRNA synthetase (EPRS), opsin (OP) and phosphoenolpyruvate carboxykinase (PEPCK) were proven to be suitable to amplify specific nucleotide fragments from selected amphipod specimens; 2. Information on novel nucleotide sequences from amphipod taxa of the family Niphagidae and related outgroup taxa. Unilocus phylogenetic trees were constructed using Bayesian analysis and show relationships among selected taxa. Altogether 299 new nucleotide sequences from 92 specimens of the family Niphargidae and related outgroup amphipod taxa are deposited in GenBank (NCBI) repository and available for further use in phylogenetic analyses.

**Table:** Specifications Table

Subject area	Biology
More specific subject area	Phylogenetic analyses, relationships of amphipod crustaceans, amplification of nuclear protein-coding loci, resolving relationships, multilocus phylogenies, molecular evolution
Type of data	Tables, Figures
How data was acquired	Oligonucleotide primer construction on the basis of arthropod sequences available in GenBank by using iCODEHOP software, utilization of known oligonucleotide primers, PCR of specific homologous DNA fragments, sequencing of the PCR products, BLAST search of homologous sequences in GenBank
Data format	Analyzed
Experimental factors	NCBI database search for selection of informative protein-coding loci for phylogenetics of crustaceans, degenerate oligonucleotide primer pairs construction for amplification of selected nuclear protein-coding loci based on available nucleotide sequences in GenBank using iCODEHOP program. Isolation of DNA of the selected amphipod specimens.
Experimental features	PCR amplification, purification of the PCR products, sequencing and editing of the sequences, phylogenetic trees construction
Data source location	Specimens and DNA are deposited at the Zoological Collection, Department of Biology, Biotechnical faculty, University of Ljubljana, Slovenia (SubBio Lab Group)
Data accessibility	GenBank NCBI (Public repository): https://www.ncbi.nlm.nih.gov/nuccore/?term= MH481451:MH481531 [accn] (for EPRS) https://www.ncbi.nlm.nih.gov/nuccore/?term= MH493738:MH493813 [accn] (for Arginine Kinase) https://www.ncbi.nlm.nih.gov/nuccore/?term= MH500354:MH500407 [accn] (for Opsin) https://www.ncbi.nlm.nih.gov/nuccore/?term= MH635367:MH635408 [accn] (for PEPCK) https://www.ncbi.nlm.nih.gov/nuccore/?term= MH668918:MH668963 [accn] (for GAPDH) Supplementary material 1
Related research article	Fišer C., Sket B., Trontelj P. A phylogenetic perspective on 160 years of troubled taxonomy of Niphargus (Crustacea: Amphipoda). Zoologica Scripta 37 (2008) 6: 665–680 [1]

**Table:** 

**Value of the data** • Five nuclear protein coding loci as useful markers for phylogenetic reconstruction of amphipod family Niphargidae are reported for the first time. Data significantly contributes to the selection of available markers for phylogenetic reconstruction based on molecular traits. • Data serves as a benchmark to resolve difficult phylogenetic relationships within niphargid or among other amphipod genera. • Data on novel degenerate oligonucleotide primer pair sequences for Arginine Kinase and GAPDH as well as PCR amplification conditions enable successful amplification of these nuclear protein coding loci in variety of amphipod crustacean specimens. • 299 edited nucleotide sequences are deposited in GenBank repository and provide valuable information for inferring phylogenetic relationships among selected taxa. • Finally nucleotide sequence data for species *Niphargellus nolli* (as a representative of niphargid genus *Niphargellus*) is reported for the first time and presents significant contribution to the knowledge of phylogenetic relationships within the family Niphargidae.

## 1 Data

For amphipod crustacean family Niphargidae only a small number of universal markers have been used for phylogenetic analyses (two fragments of ribosomal 28S, ITS (internal transcribed spacer), COI (mitochondrial cytochrome oxidase I), ribosomal 12S, H2 (histone 2)) ([1], [2], [3], [4], [5]). Among them, only very short and highly conserved fragment of histone (H2) represents nuclear protein coding locus ([6], [7]). Unilocus and multilocus analyses using this limited set of markers did not provide robust framework, hence the hierarchic relationships among and within lineages remain poorly resolved ([1], [5], [7], [8]). Low-copy nuclear protein coding loci are proved to be effective markers for inferring phylogenetic relationships among groups of arthropods within or above species level ([9], [10], [11]). They provide useful information for resolving lineages where utility of traditional non-coding ribosomal DNA and mitochondrial markers does not provide effective resolution ([10]). The data presented here provides a selection of five successfully amplified specific protein-coding loci in order to provide power to phylogenetic framework and recoverage of relationships in the family Niphargidae. The nucleotide fragments may be successfully amplified in other amphipod species as well.

### 1.1 Oligonucleotide primer sequences of 5 nuclear protein-coding loci

The list of oligonucleotide primer sequences of successfully amplified nuclear protein coding markers in niphargids is presented in Table 1.

**Table 1:** Oligonucleotide primer sequences used for successful PCR amplification and sequencing of the markers, and source of information.

Marker	Name and sequence (5′to 3′) of the primer	Comment	Source
EPRS;	EPRS_1_F: CAGGAAACAGCTATGACCGARAARGARAARTTYGC EPRS_1_R:TGTAAAACGACGGCCAGTTCCCARTGRTTRAAYTTCCA		[10]
	EPRS_2_F:CTATGACCGAGAAAGAGAAGTTCGC EPRS_2_R: CAGTGGTTGAACTTCCARGCTGG	nested PCR	
ArgKin;	ArgKin_F3: CCCCTTCAACCCYTGYCTBACYGAGGC ArgKin_R3: GGVAGCTTRATRTGGACGGAGGC		This study
PEPCK;	PEPCK-F3: GAGGGCTGGCTRGCMGARCAYATG PEPCK-R3: GGMCGCATTGCRAAYGGRTCRTGCAT		[12]
OPSIN;	OPS_1_F: TGGTAYCARTWYCCICCIATGAA OPS_1_R: CCRTAIACRATIGGRTTRTA		[10]
	OPS_2_F: CCGCCGATGAAGTCGARATGGTA OPS_2_R: TTRTAIACIGCRTTIGCYTTIGCRAA	nested PCR	
GAPDH;	GAPDH_2F: GGACTACATGGTGTACATGTTYAARTWYGA GAPDH_2R: GAGTAGCCGAACTCGTTRTCRTACCA		This study

### 1.2 PCR amplification conditions for selected markers

For marker EPRS the conditions of touchdown cycling protocol for amplification are as follows:

Initial denaturation step of 4 min at 94 °C was followed by 24 cycles of touchdown PCR. In each cycle denaturation step of 45 sec at 94 °C was followed by annealing step of 45 sec where annealing temperature decreased in increments of 0,4 °C for every subsequent set of cycles. Hence the annealing temperature of the first cycle was 55 °C and the temperature of the last cycle was 45,6 °C. The extension step of each cycle was performed at 72 °C and lasted for 1 min 30 sec. 15 cycles of denaturation of 45 sec at 94 °C, annealing step of 45 sec at 45 °C, and extension step of 1 min 30 sec at 72 °C followed. Final extension step lasted for 3 min at 72 °C.

For marker PEPCK the conditions of amplification were as follows: Initial denaturation step of 3 min at 94 °C was followed by 40 cycles of denaturation step of 45 sec at 95 °C, annealing step of 45 sec at 57 °C and extension step of 1 min at 72 °C. Final extension step lasted for 7 min at 72 °C.

For markers ArgKin, OPSIN and GAPDH the conditions of touchdown cycling protocol for amplification are as follows: Initial denaturation step of 7 min at 95 °C was followed by 25 cycles of touchdown PCR. In each cycle denaturation step of 30 sec at 95 °C was followed by annealing step of 1min where annealing temperature decreased in increments of 0,4 °C for every subsequent set of cycles. Hence the annealing temperature of the first cycle was 60 °C and the temperature of the last cycle was 50 °C. The extension step of each cycle was performed at 72 °C and lasted for 2 min. 20 cycles of denaturation of 45 sec at 94 °C, annealing step of 45 sec at 45 °C, and extension step of 1 min 30 sec at 72 °C followed. Final extension step lasted for 3 min at 72 °C.

In some cases, first amplification did not yield proper amount of the product to be used for sequencing. In this case, the second amplification using nested primer pair was performed. For nested primer pairs 1–2 μL of the product of PCR amplification was used as a template for the second amplification using nested primer pairs with the same amplification conditions.

### 1.3 New molecular sequence data and phylogenetic trees

Information on new molecular sequence datasets of protein-coding markers which were successfully amplified in specimens of the family Niphargidae and in some related amphipod crustacean taxa for the first time is presented in Table 2. Nucleotide sequences may be retrieved from GenBank repository. Additional information regarding specimens is presented in the supplementary material 1. All the newly obtained sequences were validated by BLAST searches (https://blast.ncbi.nlm.nih.gov/Blast.cgi) using optimization either for megablast or discontiguous megablast. BLAST results for each sequence obtained from the first hit are presented in the supplementary material 2. For further validation purposes all the sequences were translated into amino acids, checked for the presence of stop codons and used in alignment generation and phylogeny reconstruction.

**Table 2:** Numbers of successfully amplified sequences, fragment length, best substitution model and GenBank repository accession numbers.

Nuclear marker	Number of sequences	Fragment length (bp)	Best substitution model	GenBank repository accession numbers
EPRS	82	403	GTR+G+I	MH481451 - MH481531
Arginine Kinase	76	411	GTR+G+I	MH493738 - MH493813
PEPCK	54	633	HKY+G+I	MH500354 - MH500407
Opsin	42	737	GTR+G+I	MH635367 - MH635408
GAPDH	46	790	GTR+G+I	MH668918 - MH668963

**Fig. 1:**
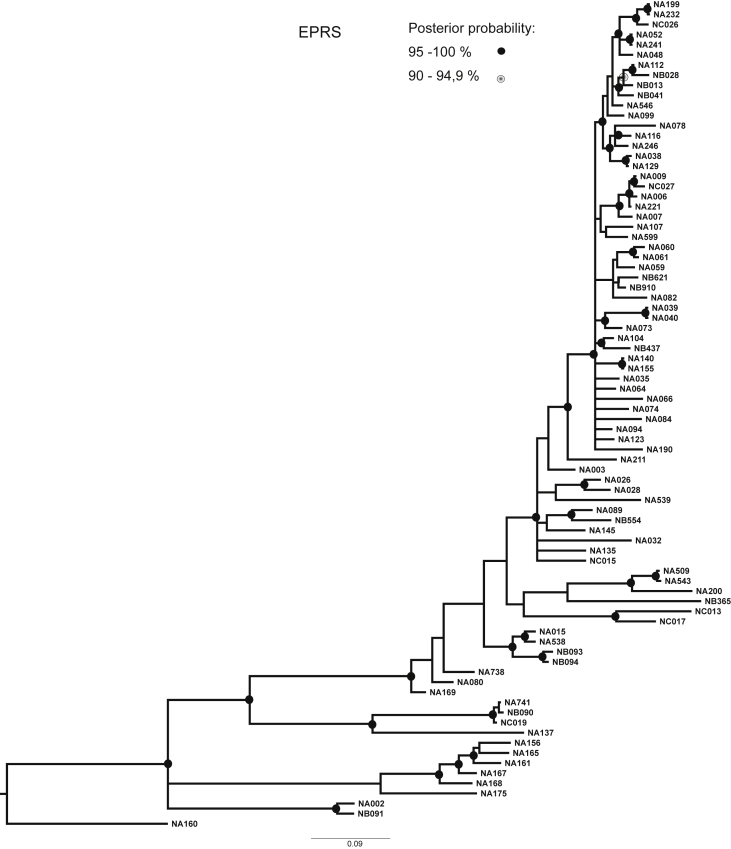
Consensus phylogenetic tree inferred by Bayesian Analysis based on EPRS marker. Posterior probabilities larger than 90 % are indicated on nodes as black or grey circles. Voucher numbers are indicated on leaves – information regarding specimens is presented in supplementary material 1.

**Fig. 2:**
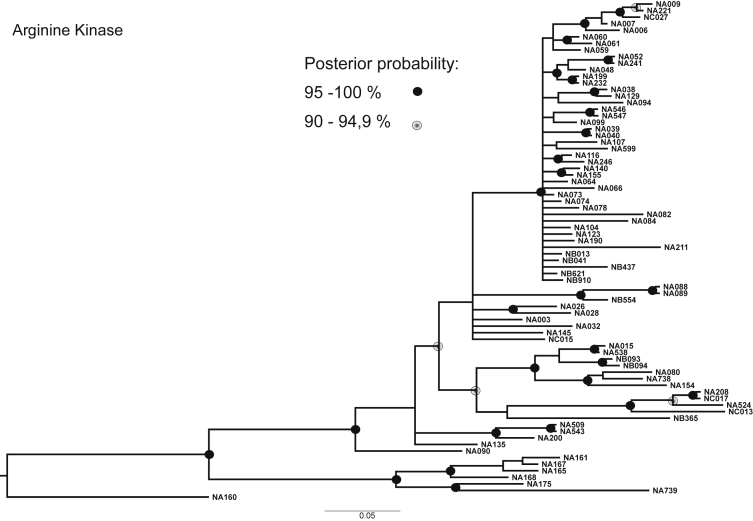
Consensus phylogenetic tree inferred by Bayesian Analysis based on ArgKin marker. Posterior probabilities larger than 90 % are indicated on nodes as black or grey circles. Voucher numbers are indicated on leaves – information regarding specimens is presented in supplementary material 1.

**Fig. 3:**
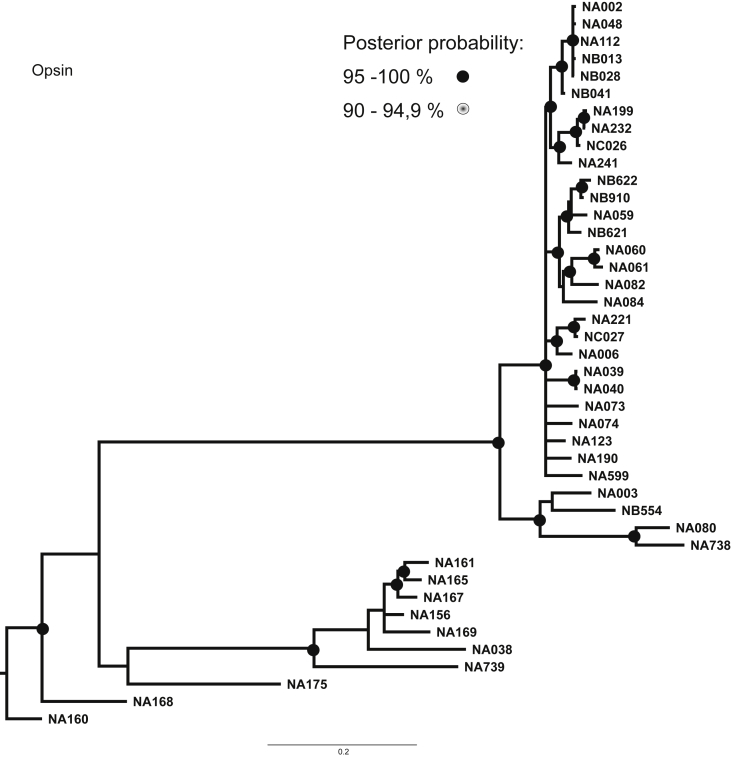
Consensus phylogenetic tree inferred by Bayesian Analysis based on Opsin marker. Posterior probabilities larger than 90 % are indicated on nodes as black or grey circles. Voucher numbers are indicated on leaves – information regarding specimens is presented in supplementary material 1.

**Fig. 4:**
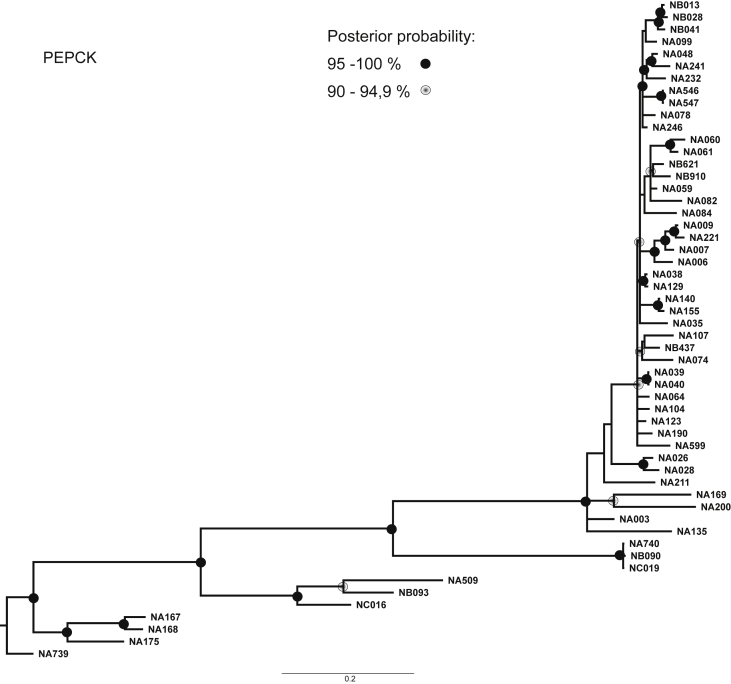
Consensus phylogenetic tree inferred by Bayesian Analysis based on PEPCK marker. Posterior probabilities larger than 90 % are indicated on nodes as black or grey circles. Voucher numbers are indicated on leaves – information regarding specimens is presented in supplementary material 1.

**Fig. 5:**
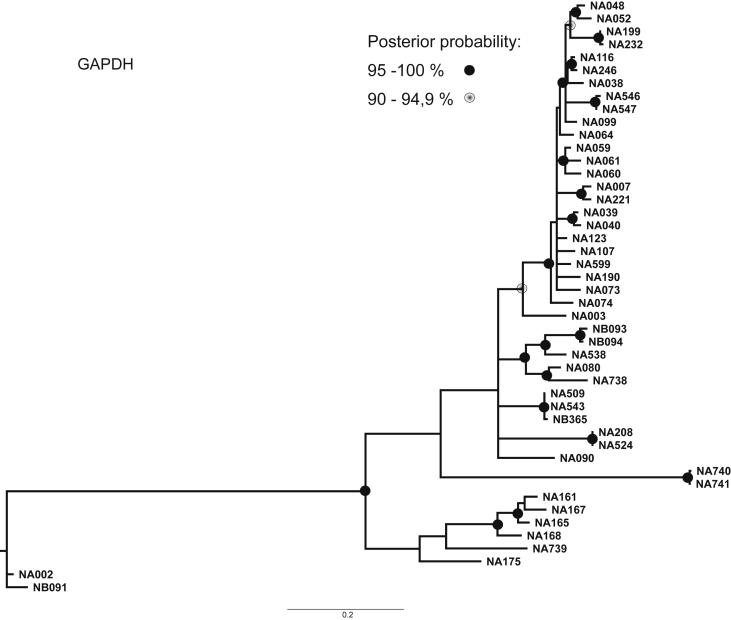
Consensus phylogenetic tree inferred by Bayesian Analysis based on GAPDH marker. Posterior probabilities larger than 90 % are indicated on nodes as black or grey circles. Voucher numbers are indicated on leaves – information regarding specimens is presented in supplementary material 1.

Phylogenetic trees for each marker were constructed using Bayesian Analysis and are shown in Fig. 1, Fig. 2, Fig. 3, Fig. 4, Fig. 5.

## 2 Experimental design, materials and methods

### 2.1 Materials

The specimens of family Niphargidae and related amphipod crustaceans were collected in time period of the last two decades. For detailed information regarding the specimens and their locality see information in supplementary material 1. Specimens for morphological analyses and isolated DNA are deposited at Zoological collection, Department of Biology, Biotechnical faculty, University of Ljubljana, Slovenia (SubBio Lab Group).

### 2.2 Search for suitable markers and existing oligonucleotide primer sequences

Information regarding suitable nuclear protein coding markers for amphipod family Niphargidae was obtained from available research literature and public databases of nucleotide sequences (GenBank, Ensembl, UniProtKB). Since no nuclear protein coding sequences for the family Niphargidae were available, the search was extended to nuclear protein-coding markers available for phylogenetic analyses in phylum Arthropoda. Selected nuclear protein coding loci were tested for successful amplification using already available oligonucleotide primers and amplification protocols. Among them, 3 markers proved to be suitable for amplification from majority of studied specimens: Glutamyl and prolyl t-RNA (EPRS), opsin and phosphoenole pyruvate charboxylase (PEPCK).

### 2.3 Oligonucleotide primer sequence pair construction

For the two housekeeping genes Arginine kinase (ArgKin) and glyceraldehyde phosphate dehydrogenase (GAPDH) we constructed new degenerate oligonucleotide primer pairs. Using the online tool BLAST (https://blast.ncbi.nlm.nih.gov/Blast.cgi) we obtained homologous sequences of several representatives of the phylum Arthropoda. We aligned nucleotide sequences using plug-in software MAFFT v. 6 implemented in Geneious Pro 5.6 (Biomatters, New Zealand) [13]. The alignment of sequences translated into amino acids was constructed using Clustal W [14]. Both alignments were used to construct degenerate oligonucleotide primer pairs for amplification of partial fragments of ArgKin and GAPDH using software iCODEhop [15].

### 2.4 DNA isolation

Entire specimen or an appendage was used for isolation of DNA. DNA was isolated using GenElute Mammalian Genomic DNA Miniprep Kit (Sigma Aldrich, USA) following the protocol for DNA isolation from tissues » Mammalian Tissue Preparation«. One specimen (*Niphargellus nolli*; voucher number NB365) was fixed in formalin. Therefore for the successful amplification of DNA we followed the protocol for DNA isolation from formalin-fixed samples [16].

### 2.5 PCR amplification, purification of the products and sequencing

The PCR amplifications were conducted in a 15-μL reaction mixture as in Ref. [8]. PCR cycling protocols followed conditions in subsection 1.2. PCR products were purified using Exonuclease I and shrimp alkaline phosphatase (Thermo Fisher Scientific, USA) as in Ref. [8]. Each fragment was sequenced in both directions using PCR amplifications primers by Macrogen Europe (Amsterdam, Netherlands).

### 2.6 Editing of the sequences

Chromatograms were assembled and sequences were edited manually using Geneious R8.1.6. and 11.1.2 [13]. Alignments of nucleotide sequences for each marker were performed using plug-in software ClustalW [14] implemented in Geneious R8.1.6 [13]. The alignments were translated into amino acids and checked for stop codons and inconsistencies. All the new sequences were submitted to GenBank repository (NCBI) (accession numbers in Table 2 and in Supplementary material 1).

### 2.7 Phylogenetic trees

The best substitution model for each marker was calculated based on Akaike information criterion (AIC) using SMS – Smart model selection on web server: http://www.atgc-montpellier.fr/phyml-sms/ [17] (Table 2). Unilocus phylogenetic trees were estimated by Bayesian analysis using MrBayes 3.2.2 [18] on the Cipres Science Gateway v 3.3. (http://www.phylo.org/index.php). Two simultaneuous runs with four chains each were run for three to four million generations until both runs reached convergence. Runs were sampled every 1000th generation. First 25 % of the sampled trees were discarded as burnin and the consensus tree of each marker was constructed by 50 % majority rule. The trees were visualised in FigTree v.1.4.3 software (http://tree.bio.ed.ac.uk/software/figtree/).
