# Supplementary material for: Five nuclear protein-coding markers for establishing a robust phylogenetic framework of niphargid crustaceans (Niphargidae: Amphipoda) and new molecular sequence data
Source: Data Brief. 2019 Jun 12;25:104134. doi: 10.1016/j.dib.2019.104134 (PMC6598839; doi:10.1016/j.dib.2019.104134)
Supplement: Multimedia component 1 [file mmc1.pdf]

Supplementary material 1

List of specimens used in this study, locality and online repository information

Family	Species	Vaucher	Locality	Country	GenBank accession numbers of markers: EPRS	ArgKin	PEPCK	OPSIN	GAPDH
Crangonyctidae	Synurella ambulans	NA002	Vode v gozdičku za stavbo NIB, pri Biološkem središču	Slovenia	MH481451	na	na	MH635367	MH668918
Niphargidae	Niphargus virei	NA003	prov. Limburg, Gulpen, well at Dorpstraat 7, Reijmerstok, Amertsfoort coordinates: 188.0; 313.0. NED	Netherlands	MH481452	MH493738	MH500354	MH635368	MH668919
Niphargidae	Niphargus sp.	NA006	Retec izvir, Cres	Croatia	na	MH493739	MH500355	MH635369	na
Niphargidae	Niphargus longicaudatus	NA007	potok ob cesti Monte Faito-Vico Equense, Casola, Napoli	Italy	MH481453	MH493740	MH500356	na	MH668920
Niphargidae	Niphargus sp.	NA009	izvir pri Lauro, Gargano	Italy	MH481454	MH493741	MH500357	na	na
Niphargidae	Niphargus wolfi	NA015	Križna jama	Slovenia	MH481455	MH493742	na	na	na
Niphargidae	Niphargus scopicauda	NA026	Huda luknja pri Gornjem Doliču	Slovenia	MH481456	MH493743	MH500358	na	na
Niphargidae	Niphargus tatrensis	NA028	Lodowe zrodlo (Icy spring), Poland	Poland	MH481457	MH493744	MH500359	na	na
Niphargidae	Niphargus schellenbergi	NA032	prov. Luxembourg, Durbuy, shallow well just along road 800 m SE of church Heyd, Lambert coordinates 235.18, 115.45, alt. 320 m	Belgium	MH481458	MH493745	na	na	na
Niphargidae	Niphargus sphagnicolus	NA035	Mostec, Rožnik, Ljubljana, Slovenia	Slovenia	MH481459	na	MH500360	na	na
Niphargidae	Niphargus hvarensis	NA038	Trsteno, Dubrovnik	Croatia	MH481460	MH493746	MH500361	MH635370	MH668921
Niphargidae	Niphargus krameri	NA039	Stranski pritok Brestovac	Croatia	MH481461	MH493747	MH500362	MH635371	MH668922
Niphargidae	Niphargus krameri	NA040	subsidiary stream of Fojba, Šestani	Croatia	MH481462	MH493748	MH500363	MH635372	MH668923
Niphargidae	Niphargus rejici	NA048	Podpeško jezero, izviri	Slovenia	MH481463	MH493749	MH500364	MH635373	MH668924
Niphargidae	Niphargus arbiter	NA052	Tounjčica cave, Ogulin	Croatia	MH481464	MH493750	na	na	MH668925
Niphargidae	Niphargus zagrebensis	NA059	Gadina, Loka	Slovenia	MH481465	MH493751	MH500365	MH635374	MH668926
Niphargidae	Niphargus dalmatinus	NA060	spring Biba, Vrana, Zadar	Croatia	MH481466	MH493752	MH500366	MH635375	MH668927
Niphargidae	Niphargus elegans	NA061	San Pancrazio	Italy	MH481467	MH493753	MH500367	MH635376	MH668928
Niphargidae	Niphargus lessiniensis	NA064	Grotta dell Aqua, Ponte de Veja, Monti Lessini,Verona	Italy	MH481468	MH493754	MH500368	na	MH668929
Niphargidae	Niphargus puteanus	NA066	Gasthof Zur Walba, izvir S od gostišča	Germany	MH481469	MH493755	na	na	na
Niphargidae	Niphargus caspary	NA073	Tuebingen	Germany	MH481470	MH493756	na	MH635377	MH668930
Niphargidae	Niphargus costozzae	NA074	Covolo della Guerra	Italy	MH481471	MH493757	MH500369	MH635378	MH668931
Niphargidae	Niphargus factor	NA078	Vjetrenica pri Zavali	Bosnia and Herzegovina	MH481472	MH493758	MH500370	na	na

Niphargidae	Niphargus grandii	NA080	Torre, Ruda, Monfalcone	Italy	MH481473	MH493759	na	MH635379	MH668932
Niphargidae	Niphargus hadzii	NA082	Izvir pod orehom, Verd, Vrhnika	Slovenia	MH481474	MH493760	MH500371	MH635380	na
Niphargidae	Niphargus illidzensis	NA084	source de la Bosna	Bosnia and Herzegovina	MH481475	MH493761	MH500372	MH635381	na
Niphargidae	Niphargus kieferi	NA088	Biberach	Germany	na	MH493762	na	na	na
Niphargidae	Niphargus kieferi	NA089	Biberach	Germany	MH481476	MH493763	na	na	na
Niphargidae	Niphargus kochianus	NA090	St Albans, Hertfordshire	United Kingdom	na	MH493764	na	na	MH668933
Niphargidae	Niphargus lourensis	NA094	Izvir reke Louros	Greece	MH481477	MH493765	na	na	na
Niphargidae	Niphargus orcinus	NA099	Križna jama	Slovenia	MH481478	MH493766	MH500373	na	MH668934
Niphargidae	Niphargus rhenorhodanensis	NA104	grotte Cormoran,Torcieu, Albarine basin, Lyon	France	MH481479	MH493767	MH500374	na	na
Niphargidae	Niphargus spinulifemur	NA107	Potok SV od Hrastovelj	Slovenia	MH481480	MH493768	MH500375	na	MH668935
Niphargidae	Niphargus subtypicus	NA112	Jama pod gradom Luknja	Slovenia	MH481481	na	na	MH635382	na
Niphargidae	Niphargus vjetrenicensis	NA116	Vjetrenica pri Zavali	Bosnia and Herzegovina	MH481482	MH493769	na	na	MH668936
Niphargidae	Niphargus stygius	NA123	Predjamski sistem	Slovenia	MH481483	MH493770	MH500376	MH635383	MH668937
Niphargidae	Niphargus hvarensis	NA129	Izvir nad vasjo Mlini	Croatia	MH481484	MH493771	MH500377	na	na
Niphargidae	Niphargus laisi	NA135	Lorrach	Germany	MH481485	MH493772	MH500378	na	na
Pseudoniphargidae	Pseudoniphargus sp.	NA137	Rupine, bentos	Croatia	MH481486	na	na	na	na
Niphargidae	Niphargus dobrogicus	NA140	Well at S Limanu, Mangalia, Dobrogea	Romania	MH481487	MH493773	MH500379	na	na
Niphargidae	Niphargus gallicus	NA145	zalit izkop ob Limanu, Hagieni, Mangalia, Dobrogea	Romania	MH481488	MH493774	na	na	na
Niphargidae	Niphargus decui	NA154	Izvir ob Limanu	Romania	na	MH493775	na	na	na
Niphargidae	Niphargus tauricus	NA155	Partizanski izvir - jamica, jugo-vzhodno od Čortove lestnice, ob stari cesto do Jalte	Ukraine	MH481489	MH493776	MH500380	na	na
Gammaridae	Pontogammarus abbreviatus	NA156	L.Razim, Jurilovka, Babadag	Romania	MH481490	na	na	MH635384	na
Hadziidae	Hadzia sp.	NA160	Izvor-spilja kod kapele Sv. Mihovila, Kosa	Croatia	MH481491	MH493777	na	MH635385	na
Gammaridae	Dikerogammarus villosus	NA161	jezero Razim, plitvina, Dobrogea	Romania	MH481492	MH493778	na	MH635386	MH668938
Pontogammaridae	Pontogammarus crassus	NA165	L.Razim, Jurilovka, Babadag	Romania	MH481493	MH493779	na	MH635387	MH668939
Pontogammaridae	Niphargoides spinicaudatus	NA167	Dunarea-Donava, Sf.Gheorghe, Murighiol, Tulcea	Romania	MH481494	MH493780	MH500381	MH635388	MH668940
Gammaridae	Chaetogammarus tenellus	NA168	L.Razim, Jurilovka, Babadag	Romania	MH481495	MH493781	MH500382	MH635389	MH668941

Niphargidae	Niphargus multipennatus	NA169	Tomačevao - intersticial	Slovenia	MH481496	na	MH500383	MH635390	na
Gammaridae	Typhlogammarus mrazeki	NA175	Vjetrenica pri Zavali	Bosnia and Herzegovina	MH481497	MH493782	MH500384	MH635391	MH668942
Niphargidae	Niphargus timavi	NA190	Izvir Postenjška	Italy	MH481498	MH493783	MH500385	MH635392	MH668943
Niphargidae	Niphargus hebereri	NA199	Jama na Bijaki, Brač	Croatia	MH481499	MH493784	na	MH635393	MH668944
Niphargidae	Niphargus angelieri	NA200	Grotte des Fees, Leucate, Perpignan	France	MH481500	MH493785	MH500386	na	na
Niphargidae	Niphargus glenniei	NA208	Reed's Cave, South Devon	United Kingdom	na	MH493786	na	na	MH668945
Niphargidae	Niphargus valachicus	NA211	Topolovka, jarek-potok, Lug Draganički, Karlovac	Croatia	MH481501	MH493787	MH500387	na	na
Niphargidae	Niphargus pasquinii	NA221	Sorgenti di Peschiera, reka 1 km od izvira, Terme di cotilia, Rieti	Italy	MH481502	MH493788	MH500388	MH635394	MH668946
Niphargidae	Niphargus hebereri	NA232	Bac jama, Split	Croatia	MH481503	MH493789	MH500389	MH635395	MH668947
Niphargidae	Niphargus arbiter	NA241	Tounjčica cave, Ogulin	Croatia	MH481504	MH493790	MH500390	MH635396	na
Niphargidae	Niphargus balcanicus	NA246	Vjetrenica pri Zavali	Bosnia and Herzegovina	MH481505	MH493791	MH500391	na	MH668948
Niphargidae	Haploginglymus sp.	NA509	Arga; prodišče, Akerreta, Pamplona	Spain	MH481506	MH493792	MH500392	na	MH668949
Niphargidae	Niphargus glenniei	NA524	North Kenwood, spring collecting chamber	United Kingdom	na	MH493793	na	na	MH668950
Niphargidae	Niphargus fongi	NA538	Dolga jama pri Koblarjih	Slovenia	MH481507	MH493794	na	na	MH668951
Niphargidae	Niphargus kochianus	NA539	na	na	MH481508	na	na	na	na
Niphargidae	Haploginglymus sp.	NA543	Arga; prodišče, Akerreta, Pamplona	Spain	MH481509	MH493795	na	na	MH668952
Niphargidae	Niphargobates orophobata	NA546	Planinska jama, Planina	Slovenia	MH481510	MH493796	MH500393	na	MH668953
Niphargidae	Niphargobates orophobata	NA547	Planina grotte, Kačja vas	Slovenia	na	MH493797	MH500394	na	MH668954
Niphargidae	Niphargus stochi	NA599	Jama pod Krogom	Slovenia	MH481511	MH493798	MH500395	MH635397	MH668955
Niphargidae	Carinurella paradoxa	NA738	Torre, S od Ruda, pod mostom	Italy	MH481512	MH493799	na	MH635398	MH668956
Gammaridae	Gammarus fossarum	NA739	Stream in proximity of Biology department, Ljubljana	Slovenia	na	MH493800	MH500396	MH635399	MH668957
Niphargidae	Microniphargus leruthi	NA740	jama Sweetwater Pot, South Devon	United Kingdom	na	na	MH500397	na	MH668958
Niphargidae	Microniphargus leruthi	NA741	jama Sweetwater Pot, South Devon	United Kingdom	MH481513	na	na	na	MH668959
Niphargidae	Niphargus croaticus	NB013	Izvor Zagorske Mrežnice, Ogulin	Croatia	MH481514	MH493801	MH500398	MH635400	na
Niphargidae	Niphargus subtypicus	NB028	Jazbina jama, Ogulin	Croatia	MH481515	na	MH500399	MH635401	na
Niphargidae	Niphargus steueri	NB041	Bunar uz Vodnjansko cesto, polje Ližnjemore, Pula	Croatia	MH481516	MH493802	MH500400	MH635402	na

Niphargidae	Microniphargus leruthi	NB090	grotte de Comblain au-Pont, Petit Lac	United Kingdom	MH481517	na	MH500401	na	na
Crangonycidae	Synurella sp.	NB091	Naravni izvir pri hiši Čagona	Slovenia	MH481518	na	na	na	MH668960
Niphargidae	Pontoniphargus ruffoi	NB093	Hagieni surface sulphurous Spring, 10 km W Movile	Romania	MH481519	MH493803	MH500402	na	MH668961
Niphargidae	Pontoniphargus racovitzai	NB094	Str. Tonitza #1	Romania	MH481520	MH493804	na	na	MH668962
Niphargidae	Niphargellus nolli	NB365	na	Germany	MH481521	MH493805	na	na	MH668963
Niphargidae	Niphargus rhenorhodanensis	NB437	Vendline, Vendlincourt source	Switzerland	MH481522	MH493806	MH500403	na	na
Niphargidae	Niphargus molnari	NB554	Abaligeti cave	Hungary	MH481523	MH493807	na	MH635403	na
Niphargidae	Niphargus brachytelson	NB621	Vančeva jama, Koblarji	Slovenia	MH481524	MH493808	MH500404	MH635404	na
Niphargidae	Niphargus novomestanus	NB622	Krška jama	Slovenia	na	na	na	MH635405	na
Niphargidae	Niphargus podpecanus	NB910	Lučka jama	Slovenia	MH481525	MH493809	MH500405	MH635406	na
Niphargidae	Niphargus irlandicus	NC013	Doolin River Cave	Ireland	MH481526	MH493810	na	na	na
Niphargidae	Niphargus wexfordensis	NC015	Polldubh, Gallway	Ireland	MH481527	MH493811	na	na	na
Niphargidae	Niphargus glenniei	NC016	North Kenwood, spring collecting chamber, Devon	United Kingdom	na	na	MH500406	na	na
Niphargidae	Niphargus glenniei	NC017	North Kenwood, spring collecting chamber, Devon	United Kingdom	MH481528	MH493812	na	na	na
Niphargidae	Microniphargus leruthi	NC019	Polldubh	Ireland	MH481529	na	MH500407	na	na
Niphargidae	Niphargus ictus	NC026	Frassasi cave system	Italy	MH481530	na	na	MH635407	na
Niphargidae	Niphargus frasassianus	NC027	Frassasi cave system	Italy	MH481531	MH493813	na	MH635408	na
