# Supplementary material for: Five nuclear protein-coding markers for establishing a robust phylogenetic framework of niphargid crustaceans (Niphargidae: Amphipoda) and new molecular sequence data
Source: Data Brief. 2019 Jun 12;25:104134. doi: 10.1016/j.dib.2019.104134 (PMC6598839; doi:10.1016/j.dib.2019.104134)
Supplement: Multimedia component 2 [file mmc2.pdf]

Supplementary material 2

BLAST results for each sequence, obtained from the first hit sequence

1. EPRS

Family	Species	Vaucher	EPRS – GenBank acc. number	1st BLAST hit description	Max Score	Total Score	Query cover	E value	% identity	Accession number
Crangonyctidae	Synurella ambulans	NA002	MH481451	PREDICTED: Penaeus vannamei bifunctional glutamate/proline--tRNA ligase-like (LOC113826345), mRNA	299	299	99%	8,00E-77	76.37%	XM_027379217.1
Niphargidae	Niphargus virei	NA003	MH481452	Cosmonotus grayi putative glutamyl-prolyl tRNA synthetase (EPRS) gene, partial cds	257	257	89%	2,00E-64	76.18%	EU910122.1
Niphargidae	Niphargus longicaudatus	NA007	MH481453	Cosmonotus grayi putative glutamyl-prolyl tRNA synthetase (EPRS) gene, partial cds	269	269	89%	4,00E-68	76.73%	EU910122.1
Niphargidae	Niphargus sp.	NA009	MH481454	Cosmonotus grayi putative glutamyl-prolyl tRNA synthetase (EPRS) gene, partial cds	238	238	87%	2,00E-58	77.89%	EU910122.1
Niphargidae	Niphargus wolfi	NA015	MH481455	Cosmonotus grayi putative glutamyl-prolyl tRNA synthetase (EPRS) gene, partial cds	282	282	90%	6,00E-72	77.22%	EU910122.1
Niphargidae	Niphargus scopicauda	NA026	MH481456	Libinia emarginata voucher LemMALA glu- + pro-tRNA synthetase mRNA, partial cds	250	250	76%	4,00E-62	77.85%	GQ887896.1
Niphargidae	Niphargus tatrensis	NA028	MH481457	Libinia emarginata voucher LemMALA glu- + pro-tRNA synthetase mRNA, partial cds	243	243	76%	6,00E-60	77.52%	GQ887896.1
Niphargidae	Niphargus schellenbergi	NA032	MH481458	Calappa gallus putative glutamyl-prolyl tRNA synthetase (EPRS) gene, partial cds	227	227	75%	4,00E-55	78.06%	EU910117.1
Niphargidae	Niphargus sphagnicolus	NA035	MH481459	Cosmonotus grayi putative glutamyl-prolyl tRNA synthetase (EPRS) gene, partial cds	251	251	86%	1,00E-62	77.99%	EU910122.1
Niphargidae	Niphargus hvarensis	NA038	MH481460	Cosmonotus grayi putative glutamyl-prolyl tRNA synthetase (EPRS) gene, partial cds	257	257	86%	2,00E-64	76.64%	EU910122.1
Niphargidae	Niphargus krameri	NA039	MH481461	Cosmonotus grayi putative glutamyl-prolyl tRNA synthetase (EPRS) gene, partial cds	260	260	89%	7,00E-65	76.52%	EU910122.1
Niphargidae	Niphargus krameri	NA040	MH481462	Cosmonotus grayi putative glutamyl-prolyl tRNA synthetase (EPRS) gene, partial cds	260	260	91%	7,00E-65	76.52%	EU910122.1
Niphargidae	Niphargus rejici	NA048	MH481463	Cosmonotus grayi putative glutamyl-prolyl tRNA synthetase (EPRS) gene, partial cds	268	268	82%	1,00E-67	78.31%	EU910122.1
Niphargidae	Niphargus arbiter	NA052	MH481464	Cosmonotus grayi putative glutamyl-prolyl tRNA synthetase (EPRS) gene, partial cds	250	250	82%	4,00E-62	77.11%	EU910122.1
Niphargidae	Niphargus zagrebensis	NA059	MH481465	Cosmonotus grayi putative glutamyl-prolyl tRNA synthetase (EPRS) gene, partial cds	265	265	89%	2,00E-66	76.45%	EU910122.1
Niphargidae	Niphargus dalmatinus	NA060	MH481466	Lepidophthalmus louisianensis putative glutamyl-prolyl tRNA synthetase (EPRS) gene, partial cds	260	260	99%	7,00E-65	74.81%	EU910104.1
Niphargidae	Niphargus elegans	NA061	MH481467	Cosmonotus grayi putative glutamyl-prolyl tRNA synthetase (EPRS) gene, partial cds	261	261	89%	2,00E-65	76.45%	EU910122.1
Niphargidae	Niphargus lessiniensis	NA064	MH481468	Cosmonotus grayi putative glutamyl-prolyl tRNA synthetase (EPRS) gene, partial cds	263	263	89%	6,00E-66	76.45%	EU910122.1
Niphargidae	Niphargus puteanus	NA066	MH481469	Cosmonotus grayi putative glutamyl-prolyl tRNA synthetase (EPRS) gene, partial cds	241	241	85%	2,00E-59	75.72%	EU910122.1
Niphargidae	Niphargus caspary	NA073	MH481470	Cosmonotus grayi putative glutamyl-prolyl tRNA synthetase (EPRS) gene, partial cds	266	266	89%	5,00E-67	76.73%	EU910122.1

Niphargidae	Niphargus costozzae	NA074	MH481471	Cosmonotus grayi putative glutamyl-prolyl tRNA synthetase (EPRS) gene, partial cds	266	266	89%	5,00E-67	76.73%	EU910122.1
Niphargidae	Niphargus factor	NA078	MH481472	Cosmonotus grayi putative glutamyl-prolyl tRNA synthetase (EPRS) gene, partial cds	238	238	85%	2,00E-58	77.04%	EU910122.1
Niphargidae	Niphargus grandii	NA080	MH481473	Praebebalia longidactyla putative glutamyl-prolyl tRNA synthetase (EPRS) gene, partial cds	240	240	73%	7,00E-59	78.28%	EU910119.1
Niphargidae	Niphargus hadzii	NA082	MH481474	Cosmonotus grayi putative glutamyl-prolyl tRNA synthetase (EPRS) gene, partial cds	260	260	89%	2,00E-65	76.45%	EU910122.1
Niphargidae	Niphargus illidzensis	NA084	MH481475	Cosmonotus grayi putative glutamyl-prolyl tRNA synthetase (EPRS) gene, partial cds	266	266	89%	5,00E-67	76.73%	EU910122.1
Niphargidae	Niphargus kieferi	NA089	MH481476	Cosmonotus grayi putative glutamyl-prolyl tRNA synthetase (EPRS) gene, partial cds	258	258	85%	2,00E-64	77.81%	EU910122.1
Niphargidae	Niphargus lourensis	NA094	MH481477	Cosmonotus grayi putative glutamyl-prolyl tRNA synthetase (EPRS) gene, partial cds	261	261	89%	2,00E-65	76.45%	EU910122.1
Niphargidae	Niphargus orcinus	NA099	MH481478	Cosmonotus grayi putative glutamyl-prolyl tRNA synthetase (EPRS) gene, partial cds	266	266	83%	5,00E-67	77.98%	EU910122.1
Niphargidae	Niphargus rhenorhodanensis	NA104	MH481479	Cosmonotus grayi putative glutamyl-prolyl tRNA synthetase (EPRS) gene, partial cds	264	264	89%	2,00E-66	76.67%	EU910122.1
Niphargidae	Niphargus spinulifemur	NA107	MH481480	Cosmonotus grayi putative glutamyl-prolyl tRNA synthetase (EPRS) gene, partial cds	257	257	89%	2,00E-64	76.18%	EU910122.1
Niphargidae	Niphargus subtypicus	NA112	MH481481	Cosmonotus grayi putative glutamyl-prolyl tRNA synthetase (EPRS) gene, partial cds	257	257	86%	2,00E-64	76.64%	EU910122.1
Niphargidae	Niphargus vjetrenicensis	NA116	MH481482	Cosmonotus grayi putative glutamyl-prolyl tRNA synthetase (EPRS) gene, partial cds	257	257	86%	2,00E-64	76.64%	EU910122.1
Niphargidae	Niphargus stygius	NA123	MH481483	Cosmonotus grayi putative glutamyl-prolyl tRNA synthetase (EPRS) gene, partial cds	240	240	89%	7,00E-59	74.79%	EU910122.1
Niphargidae	Niphargus hvarensis	NA129	MH481484	Cosmonotus grayi putative glutamyl-prolyl tRNA synthetase (EPRS) gene, partial cds	254	254	86%	3,00E-63	76.35%	EU910122.1
Niphargidae	Niphargus laisi	NA135	MH481485	Praebebalia longidactyla putative glutamyl-prolyl tRNA synthetase (EPRS) gene, partial cds	269	269	87%	1,00E-67	77.65%	EU910119.1
Pseudoniphargidae	Pseudoniphargus sp.	NA137	MH481486	Paramysis nouveli isolate 1206nouveli glutamyl-prolyl tRNA synthetase (EPRS) gene, partial cds	215	215	70%	7,00E-52	78.82%	EU233585.1
Niphargidae	Niphargus dobrogicus	NA140	MH481487	Cosmonotus grayi putative glutamyl-prolyl tRNA synthetase (EPRS) gene, partial cds	261	261	89%	2,00E-65	76.45%	EU910122.1
Niphargidae	Niphargus gallicus	NA145	MH481488	Cosmonotus grayi putative glutamyl-prolyl tRNA synthetase (EPRS) gene, partial cds	288	288	90%	1,00E-73	77.84%	EU910122.1
Niphargidae	Niphargus tauricus	NA155	MH481489	Cosmonotus grayi putative glutamyl-prolyl tRNA synthetase (EPRS) gene, partial cds	261	261	89%	2,00E-65	76.45%	EU910122.1
Gammaridae	Pontogammarus abbreviatus	NA156	MH481490	Ethusa sp. AT-2008 putative glutamyl-prolyl tRNA synthetase (EPRS) gene, partial cds	271	271	98%	1,00E-68	75.50%	EU910120.1
Hadziidae	Hadzia sp.	NA160	MH481491	PREDICTED: Penaeus vannamei bifunctional glutamate/proline--tRNA ligase-like (LOC113826345), mRNA	223	223	100%	5,00E-54	72.41%	XM_027379217.1
Gammaridae	Dikerogammarus villosus	NA161	MH481492	Ethusa sp. AT-2008 putative glutamyl-prolyl tRNA synthetase (EPRS) gene, partial cds	274	274	98%	3,00E-69	75.63%	EU910120.1
Pontogammaridae	Pontogammarus crassus	NA165	MH481493	Ethusa sp. AT-2008 putative glutamyl-prolyl tRNA synthetase (EPRS) gene, partial cds	281	281	98%	2,00E-71	76.25%	EU910120.1
Pontogammaridae	Niphargoides spinicaudatus	NA167	MH481494	Ethusa sp. AT-2008 putative glutamyl-prolyl tRNA synthetase (EPRS) gene, partial cds	285	285	98%	2,00E-72	76.25%	EU910120.1

Gammaridae	Chaetogammarus tenellus	NA168	MH481495	Ethusa sp. AT-2008 putative glutamyl-prolyl tRNA synthetase (EPRS) gene, partial cds	310	310	98%	5,00E-80	77.58%	EU910120.1
Niphargidae	Niphargus multipennatus	NA169	MH481496	Praebebalia longidactyla putative glutamyl-prolyl tRNA synthetase (EPRS) gene, partial cds	264	264	73%	2,00E-66	79.73%	EU910119.1
Gammaridae	Typhlogammarus mrazeki	NA175	MH481497	Ethusa sp. AT-2008 putative glutamyl-prolyl tRNA synthetase (EPRS) gene, partial cds	297	297	98%	3,00E-76	76.57%	EU910120.1
Niphargidae	Niphargus timavi	NA190	MH481498	Cosmonotus grayi putative glutamyl-prolyl tRNA synthetase (EPRS) gene, partial cds	275	275	89%	9,00E-70	77.29%	EU910122.1
Niphargidae	Niphargus hebereri	NA199	MH481499	Cosmonotus grayi putative glutamyl-prolyl tRNA synthetase (EPRS) gene, partial cds	243	243	83%	6,00E-60	76.49%	EU910122.1
Niphargidae	Niphargus angelieri	NA200	MH481500	Cyclograpsus cinereus putative glutamyl-prolyl tRNA synthetase (EPRS) gene, partial cds	257	257	75%	2,00E-64	79.02%	EU910130.1
Niphargidae	Niphargus valachicus	NA211	MH481501	Cosmonotus grayi putative glutamyl-prolyl tRNA synthetase (EPRS) gene, partial cds	257	257	85%	2,00E-64	76.88%	EU910122.1
Niphargidae	Niphargus pasquinii	NA221	MH481502	Cosmonotus grayi putative glutamyl-prolyl tRNA synthetase (EPRS) gene, partial cds	275	275	89%	9,00E-70	77.29%	EU910122.1
Niphargidae	Niphargus hebereri	NA232	MH481503	Cosmonotus grayi putative glutamyl-prolyl tRNA synthetase (EPRS) gene, partial cds	243	243	83%	6,00E-60	76.49%	EU910122.1
Niphargidae	Niphargus arbiter	NA241	MH481504	Cosmonotus grayi putative glutamyl-prolyl tRNA synthetase (EPRS) gene, partial cds	250	250	82%	4,00E-62	77.11%	EU910122.1
Niphargidae	Niphargus balcanicus	NA246	MH481505	Cosmonotus grayi putative glutamyl-prolyl tRNA synthetase (EPRS) gene, partial cds	260	260	86%	2,00E-65	77.27%	EU910122.1
Niphargidae	Haploginglymus sp.	NA509	MH481506	Cosmonotus grayi putative glutamyl-prolyl tRNA synthetase (EPRS) gene, partial cds	280	280	89%	2,00E-71	77.22%	EU910122.1
Niphargidae	Niphargus fongi	NA538	MH481507	Cosmonotus grayi putative glutamyl-prolyl tRNA synthetase (EPRS) gene, partial cds	280	280	89%	2,00E-71	77.22%	EU910122.1
Niphargidae	Niphargus kochianus	NA539	MH481508	Praebebalia longidactyla putative glutamyl-prolyl tRNA synthetase (EPRS) gene, partial cds	266	266	76%	5,00E-67	79.15%	EU910119.1
Niphargidae	Haploginglymus sp.	NA543	MH481509	Cosmonotus grayi putative glutamyl-prolyl tRNA synthetase (EPRS) gene, partial cds	280	280	89%	2,00E-71	77.22%	EU910122.1
Niphargidae	Niphargobates orophobata	NA546	MH481510	Cosmonotus grayi putative glutamyl-prolyl tRNA synthetase (EPRS) gene, partial cds	266	266	86%	5,00E-67	77.21%	EU910122.1
Niphargidae	Niphargus stochi	NA599	MH481511	Praebebalia longidactyla putative glutamyl-prolyl tRNA synthetase (EPRS) gene, partial cds	252	252	85%	1,00E-62	76.52%	EU910119.1
Niphargidae	Carinurella paradoxa	NA738	MH481512	Cosmonotus grayi putative glutamyl-prolyl tRNA synthetase (EPRS) gene, partial cds	240	240	89%	7,00E-59	74.72%	EU910122.1
Niphargidae	Microniphargus leruthi	NA741	MH481513	Ethusa sp. AT-2008 putative glutamyl-prolyl tRNA synthetase (EPRS) gene, partial cds	262	262	71%	6,00E-66	80.00%	EU910120.1
Niphargidae	Niphargus croaticus	NB013	MH481514	Cosmonotus grayi putative glutamyl-prolyl tRNA synthetase (EPRS) gene, partial cds	261	261	86%	2,00E-65	76.92%	EU910122.1
Niphargidae	Niphargus subtypicus	NB028	MH481515	Praebebalia longidactyla putative glutamyl-prolyl tRNA synthetase (EPRS) gene, partial cds	235	235	75%	8,00E-58	77.05%	EU910119.1
Niphargidae	Niphargus steueri	NB041	MH481516	Cosmonotus grayi putative glutamyl-prolyl tRNA synthetase (EPRS) gene, partial cds	252	252	86%	1,00E-62	76.35%	EU910122.1
Niphargidae	Microniphargus leruthi	NB090	MH481517	Ethusa sp. AT-2008 putative glutamyl-prolyl tRNA synthetase (EPRS) gene, partial cds	262	262	71%	6,00E-66	80.00%	EU910120.1
Crangonycidae	Synurella sp.	NB091	MH481518	PREDICTED: Penaeus vannamei bifunctional glutamate/proline--tRNA ligase-like (LOC113826345), mRNA	300	300	99%	2,00E-77	76.92%	XM_027379217.1
Niphargidae	Pontoniphargus ruffoi	NB093	MH481519	Cosmonotus grayi putative glutamyl-prolyl tRNA synthetase (EPRS) gene, partial cds	288	288	89%	1,00E-73	77.72%	EU910122.1

Niphargidae	Pontoniphargus racovitzai	NB094	MH481520	Cosmonotus grayi putative glutamyl-prolyl tRNA synthetase (EPRS) gene, partial cds	292	292	89%	1,00E-74	77.99%	EU910122.1
Niphargidae	Niphargellus nolli	NB365	MH481521	Cyclograpsus cinereus putative glutamyl-prolyl tRNA synthetase (EPRS) gene, partial cds	235	235	67%	7,00E-58	82.61%	EU910130.1
Niphargidae	Niphargus rhenorhodanensis	NB437	MH481522	Cosmonotus grayi putative glutamyl-prolyl tRNA synthetase (EPRS) gene, partial cds	257	257	89%	2,00E-64	76.18%	EU910122.1
Niphargidae	Niphargus molnari	NB554	MH481523	Cosmonotus grayi putative glutamyl-prolyl tRNA synthetase (EPRS) gene, partial cds	267	267	89%	5,00E-67	76.39%	EU910122.1
Niphargidae	Niphargus brachytelson	NB621	MH481524	Cosmonotus grayi putative glutamyl-prolyl tRNA synthetase (EPRS) gene, partial cds	275	275	89%	9,00E-70	77.29%	EU910122.1
Niphargidae	Niphargus podpecanus	NB910	MH481525	Cosmonotus grayi putative glutamyl-prolyl tRNA synthetase (EPRS) gene, partial cds	257	257	89%	2,00E-64	76.18%	EU910122.1
Niphargidae	Niphargus irlandicus	NC013	MH481526	Lepidophthalmus louisianensis putative glutamyl-prolyl tRNA synthetase (EPRS) gene, partial cds	242	242	81%	5,00E-60	76.38%	EU910104.1
Niphargidae	Niphargus wexfordensis	NC015	MH481527	Cosmonotus grayi putative glutamyl-prolyl tRNA synthetase (EPRS) gene, partial cds	279	279	89%	8,00E-71	77.56%	EU910122.1
Niphargidae	Niphargus glenniei	NC017	MH481528	Praebebalia longidactyla putative glutamyl-prolyl tRNA synthetase (EPRS) gene, partial cds	260	260	81%	6,00E-65	79.39%	EU910119.1
Niphargidae	Microniphargus leruthi	NC019	MH481529	Ethusa sp. AT-2008 putative glutamyl-prolyl tRNA synthetase (EPRS) gene, partial cds	247	247	66%	4,00E-61	81.93%	EU910120.1
Niphargidae	Niphargus ictus	NC026	MH481530	Cosmonotus grayi putative glutamyl-prolyl tRNA synthetase (EPRS) gene, partial cds	248	248	92%	1,00E-61	76.79%	EU910122.1
Niphargidae	Niphargus frasassianus	NC027	MH481531	Cosmonotus grayi putative glutamyl-prolyl tRNA synthetase (EPRS) gene, partial cds	263	263	89%	6,00E-66	76.45%	EU910122.1

2. ArgKin

Family	Species	Vaucher	ArgKin - GenBank acc. number	1st BLAST hit description	Max Score	Total Score	Query cover	E value	% identity	Accession number
Niphargidae	Niphargus virei	NA003	MH493738	Percnon affine voucher MSLKHC-BR117-Paff arginine kinase (AK) gene, partial cds	425	425	98%	8,00E-115	83.21%	KJ132743.1
Niphargidae	Niphargus sp.	NA006	MH493739	Pteromalus puparum arginine kinase mRNA, complete cds	441	441	98%	1,00E-119	84.16%	FJ882065.1
Niphargidae	Niphargus longicaudatus	NA007	MH493740	Pteromalus puparum arginine kinase mRNA, complete cds	440	440	98%	4,00E-119	83.95%	FJ882065.1
Niphargidae	Niphargus sp.	NA009	MH493741	PREDICTED: Hyalella azteca arginine kinase (LOC108677185), mRNA	443	443	99%	3,00E-120	83.90%	XM_018165356.1
Niphargidae	Niphargus wolfi	NA015	MH493742	PREDICTED: Hyalella azteca arginine kinase (LOC108677185), mRNA	409	409	99%	5,00E-110	84.67%	XM_018165356.1
Niphargidae	Niphargus scopicauda	NA026	MH493743	Glyptograpsus jamaicensis voucher SMF-25987 arginine kinase (AK) gene, partial cds	383	383	98%	3,00E-102	83.82%	KJ132692.1
Niphargidae	Niphargus tatrensis	NA028	MH493744	Lybia tessellata voucher MSLKHC-Ltes arginine kinase (AK) gene, partial cds	377	377	98%	1,00E-100	83.50%	KJ132708.1
Niphargidae	Niphargus schellenbergi	NA032	MH493745	Aulonogyrus caffer isolate GTG35 arginine kinase (AK) gene, partial cds	353	353	96%	2,00E-93	82.66%	MH030001.1
Niphargidae	Niphargus hvarensis	NA038	MH493746	Clibanarius englaucus voucher NTOU A01095 arginine kinase (AK) gene, partial cds	372	372	98%	6,00E-99	83.25%	GU382881.1
Niphargidae	Niphargus krameri	NA039	MH493747	PREDICTED: Hyalella azteca arginine kinase (LOC108677185), mRNA	387	387	99%	2,00E-103	83.74%	XM_018165356.1
Niphargidae	Niphargus krameri	NA040	MH493748	PREDICTED: Hyalella azteca arginine kinase (LOC108677185), mRNA	387	387	99%	2,00E-103	83.74%	XM_018165356.1
Niphargidae	Niphargus rejici	NA048	MH493749	Uca lactea voucher MSLKHC-BR163-Ulac arginine kinase (AK) gene, partial cds	392	392	96%	5,00E-105	84.46%	KJ132778.1
Niphargidae	Niphargus arbiter	NA052	MH493750	PREDICTED: Hyalella azteca arginine kinase (LOC108677185), mRNA	392	392	99%	5,00E-105	84.02%	XM_018165356.1
Niphargidae	Niphargus zagrebensis	NA059	MH493751	PREDICTED: Hyalella azteca arginine kinase (LOC108677185), mRNA	403	403	99%	2,00E-108	84.47%	XM_018165356.1
Niphargidae	Niphargus dalmatinus	NA060	MH493752	PREDICTED: Hyalella azteca arginine kinase (LOC108677185), mRNA	403	403	99%	2,00E-108	84.47%	XM_018165356.1
Niphargidae	Niphargus elegans	NA061	MH493753	PREDICTED: Hyalella azteca arginine kinase (LOC108677185), mRNA	411	411	99%	1,00E-110	84.71%	XM_018165356.1
Niphargidae	Niphargus lessiniensis	NA064	MH493754	Clibanarius englaucus voucher NTOU A01095 arginine kinase (AK) gene, partial cds	388	388	98%	6,00E-104	83.99%	GU382881.1
Niphargidae	Niphargus puteanus	NA066	MH493755	PREDICTED: Hyalella azteca arginine kinase (LOC108677185), mRNA	394	394	99%	1,00E-105	83.98%	XM_018165356.1
Niphargidae	Niphargus caspary	NA073	MH493756	PREDICTED: Hyalella azteca arginine kinase (LOC108677185), mRNA	398	398	99%	1,00E-106	84.22%	XM_018165356.1
Niphargidae	Niphargus costozzae	NA074	MH493757	PREDICTED: Hyalella azteca arginine kinase (LOC108677185), mRNA	409	409	99%	5,00E-110	84.71%	XM_018165356.1
Niphargidae	Niphargus factor	NA078	MH493758	Austinogebia edulis voucher MSLKHC-AeduHK arginine kinase (AK) gene, partial cds	394	394	97%	1,00E-105	84.33%	GU382902.1
Niphargidae	Niphargus grandii	NA080	MH493759	Hippa adactyla voucher NMNS 4368-027 arginine kinase (AK) gene, partial cds	427	427	98%	1,00E-115	85.64%	GU382867.1

Niphargidae	Niphargus hadzii	NA082	MH493760	PREDICTED: Hyalella azteca arginine kinase (LOC108677185), mRNA	387	387	99%	2,00E-103	83.74%	XM_018165356.1
Niphargidae	Niphargus illidzensis	NA084	MH493761	PREDICTED: Hyalella azteca arginine kinase (LOC108677185), mRNA	398	398	99%	1,00E-106	84.22%	XM_018165356.1
Niphargidae	Niphargus kieferi	NA088	MH493762	PREDICTED: Hyalella azteca arginine kinase (LOC108677185), mRNA	346	346	96%	4,00E-91	82.46%	XM_018165356.1
Niphargidae	Niphargus kieferi	NA089	MH493763	PREDICTED: Hyalella azteca arginine kinase (LOC108677185), mRNA	346	346	96%	4,00E-91	82.46%	XM_018165356.1
Niphargidae	Niphargus kochianus	NA090	MH493764	Calcinus laevimanus voucher NTOU A01100 arginine kinase (AK) gene, partial cds	436	436	97%	2,00E-118	86.28%	GU382860.1
Niphargidae	Niphargus lourensis	NA094	MH493765	PREDICTED: Hyalella azteca arginine kinase (LOC108677185), mRNA	383	383	99%	3,00E-102	83.50%	XM_018165356.1
Niphargidae	Niphargus orcinus	NA099	MH493766	Uca lactea voucher MSLKHC-BR163-Ulac arginine kinase (AK) gene, partial cds	424	424	98%	2,00E-114	85.47%	KJ132778.1
Niphargidae	Niphargus rhenorhodanensis	NA104	MH493767	PREDICTED: Hyalella azteca arginine kinase (LOC108677185), mRNA	403	403	99%	2,00E-108	84.43%	XM_018165356.1
Niphargidae	Niphargus spinulifemur	NA107	MH493768	PREDICTED: Hyalella azteca arginine kinase (LOC108677185), mRNA	420	420	99%	2,00E-113	85.19%	XM_018165356.1
Niphargidae	Niphargus vjetrenicensis	NA116	MH493769	PREDICTED: Hyalella azteca arginine kinase (LOC108677185), mRNA	387	387	99%	2,00E-103	83.74%	XM_018165356.1
Niphargidae	Niphargus stygius	NA123	MH493770	PREDICTED: Hyalella azteca arginine kinase (LOC108677185), mRNA	392	392	99%	5,00E-105	83.98%	XM_018165356.1
Niphargidae	Niphargus hvarensis	NA129	MH493771	Clibanarius englaucus voucher NTOU A01095 arginine kinase (AK) gene, partial cds	355	355	98%	7,00E-94	82.51%	GU382881.1
Niphargidae	Niphargus laisi	NA135	MH493772	Uca lactea voucher MSLKHC-BR163-Ulac arginine kinase (AK) gene, partial cds	435	435	98%	8,00E-118	85.96%	KJ132778.1
Niphargidae	Niphargus dobrogicus	NA140	MH493773	Hippa adactyla voucher NMNS 4368-027 arginine kinase (AK) gene, partial cds	394	394	97%	1,00E-105	84.33%	GU382867.1
Niphargidae	Niphargus gallicus	NA145	MH493774	Neopetrolisthes maculatus voucher NTOU A00928 arginine kinase (AK) gene, partial cds	383	383	97%	3,00E-102	83.79%	GU382876.1
Niphargidae	Niphargus decui	NA154	MH493775	Laomedia astacina voucher MSLKHC-Last arginine kinase (AK) gene, partial cds	399	399	98%	3,00E-107	84.44%	KJ132704.1
Niphargidae	Niphargus tauricus	NA155	MH493776	PREDICTED: Hyalella azteca arginine kinase (LOC108677185), mRNA	409	409	99%	5,00E-110	84.71%	XM_018165356.1
Hadziidae	Hadzia sp.	NA160	MH493777	Uca lactea voucher MSLKHC-BR163-Ulac arginine kinase (AK) gene, partial cds	411	411	100%	1,00E-110	84.67%	KJ132778.1
Gammaridae	Dikerogammarus villosus	NA161	MH493778	Crangon crangon arginine kinase mRNA, complete cds	466	466	98%	3,00E-127	87.41%	FJ457622.1
Pontogammaridae	Pontogammarus crassus	NA165	MH493779	Laomedia astacina voucher MSLKHC-Last arginine kinase (AK) gene, partial cds	444	444	98%	1,00E-120	86.42%	KJ132704.1
Pontogammaridae	Niphargoides spinicaudatus	NA167	MH493780	Penaeus monodon arginine kinase mRNA, complete cds	477	477	98%	1,00E-130	87.90%	HM034313.1
Gammaridae	Chaetogammarus tenellus	NA168	MH493781	Penaeus monodon arginine kinase mRNA, complete cds	448	448	98%	1,00E-121	86.67%	HM034313.1
Gammaridae	Typhlogammarus mrazeki	NA175	MH493782	Laomedia astacina voucher MSLKHC-Last arginine kinase (AK) gene, partial cds	433	433	98%	3,00E-117	85.93%	KJ132704.1
Niphargidae	Niphargus timavi	NA190	MH493783	PREDICTED: Hyalella azteca arginine kinase (LOC108677185), mRNA	412	412	99%	4,00E-111	84.84%	XM_018165356.1
Niphargidae	Niphargus hebereri	NA199	MH493784	Jonas distinctus voucher NTOU B00002 arginine kinase (AK) gene, partial cds	370	370	96%	2,00E-98	83.46%	KJ132701.1

Niphargidae	Niphargus angelieri	NA200	MH493785	Austinogebia edulis voucher MSLKHC-AeduHK arginine kinase (AK) gene, partial cds	427	427	97%	1,00E-115	85.79%	GU382902.1
Niphargidae	Niphargus glenniei	NA208	MH493786	Neopetrolisthes maculatus voucher NTOU A00928 arginine kinase (AK) gene, partial cds	422	422	98%	6,00E-114	85.40%	GU382876.1
Niphargidae	Niphargus valachicus	NA211	MH493787	Hydrocanthus sp. UNM KBMHcsp570 arginine kinase (ArgKin) gene, partial cds	357	357	100%	2,00E-94	82.24%	KJ548490.1
Niphargidae	Niphargus pasquinii	NA221	MH493788	PREDICTED: Hyalella azteca arginine kinase (LOC108677185), mRNA	409	409	99%	5,00E-110	84.71%	XM_018165356.1
Niphargidae	Niphargus hebereri	NA232	MH493789	Jonas distinctus voucher NTOU B00002 arginine kinase (AK) gene, partial cds	370	370	96%	2,00E-98	83.46%	KJ132701.1
Niphargidae	Niphargus arbiter	NA241	MH493790	PREDICTED: Hyalella azteca arginine kinase (LOC108677185), mRNA	390	390	99%	2,00E-104	83.78%	XM_018165356.1
Niphargidae	Niphargus balcanicus	NA246	MH493791	Clibanarius englaucus voucher NTOU A01095 arginine kinase (AK) gene, partial cds	370	370	97%	2,00E-98	83.33%	GU382881.1
Niphargidae	Haploginglymus sp.	NA509	MH493792	PREDICTED: Hyalella azteca arginine kinase (LOC108677185), mRNA	403	403	99%	2,00E-108	84.39%	XM_018165356.1
Niphargidae	Niphargus glenniei	NA524	MH493793	Neopetrolisthes maculatus voucher NTOU A00928 arginine kinase (AK) gene, partial cds	433	433	98%	3,00E-117	85.93%	GU382876.1
Niphargidae	Niphargus fongi	NA538	MH493794	PREDICTED: Hyalella azteca arginine kinase (LOC108677185), mRNA	414	414	99%	1,00E-111	84.91%	XM_018165356.1
Niphargidae	Haploginglymus sp.	NA543	MH493795	PREDICTED: Hyalella azteca arginine kinase (LOC108677185), mRNA	403	403	99%	2,00E-108	84.39%	XM_018165356.1
Niphargidae	Niphargobates orophobata	NA546	MH493796	Calosoma scrutator arginine kinase gene, partial cds	355	355	96%	7,00E-94	82.75%	EU681831.1
Niphargidae	Niphargobates orophobata	NA547	MH493797	Calosoma scrutator arginine kinase gene, partial cds	353	353	96%	2,00E-93	82.75%	EU681831.1
Niphargidae	Niphargus stochi	NA599	MH493798	PREDICTED: Hyalella azteca arginine kinase (LOC108677185), mRNA	425	425	99%	5,00E-115	85.44%	XM_018165356.1
Niphargidae	Carinurella paradoxa	NA738	MH493799	Laomedia astacina voucher MSLKHC-Last arginine kinase (AK) gene, partial cds	411	411	98%	1,00E-110	84.94%	KJ132704.1
Gammaridae	Gammarus fossarum	NA739	MH493800	Uca lactea voucher MSLKHC-BR163-Ulac arginine kinase (AK) gene, partial cds	390	390	98%	2,00E-104	84.03%	KJ132778.1
Niphargidae	Niphargus croaticus	NB013	MH493801	PREDICTED: Hyalella azteca arginine kinase (LOC108677185), mRNA	387	387	99%	2,00E-103	83.74%	XM_018165356.1
Niphargidae	Niphargus steueri	NB041	MH493802	Pteromalus puparum arginine kinase mRNA, complete cds	422	422	98%	6,00E-114	85.47%	FJ882065.1
Niphargidae	Pontoniphargus ruffoi	NB093	MH493803	PREDICTED: Hyalella azteca arginine kinase (LOC108677185), mRNA	425	425	99%	5,00E-115	85.40%	XM_018165356.1
Niphargidae	Pontoniphargus racovitzai	NB094	MH493804	PREDICTED: Hyalella azteca arginine kinase (LOC108677185), mRNA	431	431	99%	1,00E-116	85.64%	XM_018165356.1
Niphargidae	Niphargellus nolli	NB365	MH493805	Oncopagurus orientalis voucher NTOU A00371 arginine kinase (AK) gene, partial cds	398	398	97%	1,00E-106	84.54%	GU382878.1
Niphargidae	Niphargus rhenorhodanensis	NB437	MH493806	PREDICTED: Hyalella azteca arginine kinase (LOC108677185), mRNA	377	377	100%	1,00E-100	83.25%	XM_018165356.1
Niphargidae	Niphargus molnari	NB554	MH493807	Othius punctulatus voucher OTHpun01 arginine kinase (ArgK) gene, partial cds	370	370	96%	2,00E-98	83.42%	KT021883.1
Niphargidae	Niphargus brachytelson	NB621	MH493808	PREDICTED: Hyalella azteca arginine kinase (LOC108677185), mRNA	409	409	99%	5,00E-110	84.71%	XM_018165356.1
Niphargidae	Niphargus podpecanus	NB910	MH493809	PREDICTED: Hyalella azteca arginine kinase (LOC108677185), mRNA	405	405	99%	6,00E-109	84.47%	XM_018165356.1

Niphargidae	Niphargus irlandicus	NC013	MH493810	PREDICTED: Hyalella azteca arginine kinase (LOC108677185), mRNA	387	387	99%	2,00E-103	83.78%	XM_018165356.1
Niphargidae	Niphargus wexfordensis	NC015	MH493811	Uca longisignalis arginine kinase (AK) gene, partial cds	390	390	98%	2,00E-104	84.03%	EU329114.1
Niphargidae	Niphargus glenniei	NC017	MH493812	Neopetrolisthes maculatus voucher NTOU A00928 arginine kinase (AK) gene, partial cds	422	422	98%	6,00E-114	85.40%	GU382876.1
Niphargidae	Niphargus frasassianus	NC027	MH493813	PREDICTED: Hyalella azteca arginine kinase (LOC108677185), mRNA	392	392	99%	5,00E-105	83.98%	XM_018165356.1

3. PEPCK

Family	Species	Vaucher	PEPCK - GenBank acc. number	1st BLAST hit description	Max Score	Total Score	Query cover	E value	% identity	Accession number
Niphargidae	Niphargus virei	NA003	MH500354	PREDICTED: Penaeus vannamei phosphoenolpyruvate carboxykinase, cytosolic [GTP]-like (LOC113826096), transcript variant X2, mRNA	355	355	100%	2,00E-93	72,80%	XM_027378975.1
Niphargidae	Niphargus sp.	NA006	MH500355	Penaeus vannamei mRNA for phosphoenolpyruvate carboxykinase (pepck gene)	346	346	100%	1,00E-90	72,64%	AJ250829.1
Niphargidae	Niphargus longicaudatus	NA007	MH500356	Penaeus vannamei mRNA for phosphoenolpyruvate carboxykinase (pepck gene)	330	330	100%	8,00E-86	72,30%	AJ250829.1
Niphargidae	Niphargus sp.	NA009	MH500357	Macrobrachium nipponense GTP phosphoenolpyruvate carboxykinase mRNA, partial cds	336	336	95%	5,00E-88	72,70%	KP690142.1
Niphargidae	Niphargus scopicauda	NA026	MH500358	Metapenaeus brevicornis phosphoenolpyruvate carboxykinase (PEPCK) gene, partial cds	334	334	86%	6,00E-87	74,32%	JX100445.1
Niphargidae	Niphargus tatrensis	NA028	MH500359	Metapenaeus brevicornis phosphoenolpyruvate carboxykinase (PEPCK) gene, partial cds	343	343	86%	1,00E-89	73,91%	JX100445.1
Niphargidae	Niphargus sphagnicolus	NA035	MH500360	Metapenaeopsis dalei isolate MdalPEPCK2 phosphoenolpyruvate carboxykinase gene, partial cds	324	324	96%	3,00E-84	74,56%	KY864324.1
Niphargidae	Niphargus hvarensis	NA038	MH500361	PREDICTED: Penaeus vannamei phosphoenolpyruvate carboxykinase, cytosolic [GTP]-like (LOC113826097), mRNA	337	337	99%	5,00E-88	72,62%	XM_027378976.1
Niphargidae	Niphargus krameri	NA039	MH500362	PREDICTED: Penaeus vannamei phosphoenolpyruvate carboxykinase, cytosolic [GTP]-like (LOC113826097), mRNA	341	341	95%	4,00E-89	72,95%	XM_027378976.1
Niphargidae	Niphargus krameri	NA040	MH500363	PREDICTED: Penaeus vannamei phosphoenolpyruvate carboxykinase, cytosolic [GTP]-like (LOC113826097), mRNA	342	342	95%	1,00E-89	73,11%	XM_027378976.1
Niphargidae	Niphargus rejici	NA048	MH500364	PREDICTED: Penaeus vannamei phosphoenolpyruvate carboxykinase, cytosolic [GTP]-like (LOC113826097), mRNA	346	346	95%	1,00E-90	73,28%	XM_027378976.1
Niphargidae	Niphargus zagrebensis	NA059	MH500365	PREDICTED: Penaeus vannamei phosphoenolpyruvate carboxykinase, cytosolic [GTP]-like (LOC113826097), mRNA	334	334	95%	2,00E-87	72,79%	XM_027378976.1
Niphargidae	Niphargus dalmatinus	NA060	MH500366	Macrobrachium nipponense GTP phosphoenolpyruvate carboxykinase mRNA, partial cds	329	329	100%	8,00E-86	71,97%	KP690142.1
Niphargidae	Niphargus elegans	NA061	MH500367	PREDICTED: Penaeus vannamei phosphoenolpyruvate carboxykinase, cytosolic [GTP]-like (LOC113824256), misc_RNA	324	324	100%	3,00E-84	71,74%	XR_003477494.1
Niphargidae	Niphargus lessiniensis	NA064	MH500368	PREDICTED: Penaeus vannamei phosphoenolpyruvate carboxykinase, cytosolic [GTP]-like (LOC113826096), transcript variant X2, mRNA	345	345	100%	1,00E-90	72,68%	XM_027378975.1
Niphargidae	Niphargus costozzae	NA074	MH500369	PREDICTED: Penaeus vannamei phosphoenolpyruvate carboxykinase, cytosolic [GTP]-like (LOC113826097), mRNA	364	364	93%	4,00E-96	73,28%	XM_027378976.1
Niphargidae	Niphargus factor	NA078	MH500370	Metapenaeopsis mogiensis isolate MmogPEPCK2 phosphoenolpyruvate carboxykinase gene, partial cds	289	289	95%	5,00E-74	74,57%	KY864297.1
Niphargidae	Niphargus hadzii	NA082	MH500371	Macrobrachium nipponense GTP phosphoenolpyruvate carboxykinase mRNA, partial cds	333	333	95%	6,00E-87	72,79%	KP690142.1
Niphargidae	Niphargus illidzensis	NA084	MH500372	Nephrops norvegicus mRNA for Phosphoenolpyruvate carboxykinase	369	369	99%	9,00E-98	73,58%	AJ132380.1
Niphargidae	Niphargus orcinus	NA099	MH500373	Metapenaeopsis dalei isolate MdalPEPCK2 phosphoenolpyruvate carboxykinase gene, partial cds	337	337	80%	5,00E-88	75,15%	KY864324.1

Niphargidae	Niphargus rhenorhodanensis	NA104	MH500374	PREDICTED: Penaeus vannamei phosphoenolpyruvate carboxykinase, cytosolic [GTP]-like (LOC113826096), transcript variant X2, mRNA	368	368	100%	3,00E-97	73,47%	XM_027378975.1
Niphargidae	Niphargus spinulifemur	NA107	MH500375	PREDICTED: Penaeus vannamei phosphoenolpyruvate carboxykinase, cytosolic [GTP]-like (LOC113826097), mRNA	314	314	95%	6,00E-81	71,94%	XM_027378976.1
Niphargidae	Niphargus stygius	NA123	MH500376	PREDICTED: Penaeus vannamei phosphoenolpyruvate carboxykinase, cytosolic [GTP]-like (LOC113826096), transcript variant X2, mRNA	358	358	97%	2,00E-94	73,68%	XM_027378975.1
Niphargidae	Niphargus hvarensis	NA129	MH500377	PREDICTED: Penaeus vannamei phosphoenolpyruvate carboxykinase, cytosolic [GTP]-like (LOC113826097), mRNA	346	346	95%	1,00E-90	73,28%	XM_027378976.1
Niphargidae	Niphargus laisi	NA135	MH500378	PREDICTED: Penaeus vannamei phosphoenolpyruvate carboxykinase, cytosolic [GTP]-like (LOC113826096), transcript variant X2, mRNA	324	324	100%	3,00E-84	71,25%	XM_027378975.1
Niphargidae	Niphargus dobrogicus	NA140	MH500379	Penaeus vannamei mRNA for phosphoenolpyruvate carboxykinase (pepck gene)	328	328	100%	3,00E-85	72,01%	AJ250829.1
Niphargidae	Niphargus tauricus	NA155	MH500380	PREDICTED: Penaeus vannamei phosphoenolpyruvate carboxykinase, cytosolic [GTP]-like (LOC113826097), mRNA	323	323	99%	1,00E-83	72,49%	XM_027378976.1
Pontogammaridae	Niphargoides spinicaudatus	NA167	MH500381	Macrobrachium nipponense GTP phosphoenolpyruvate carboxykinase mRNA, partial cds	484	484	100%	2,00E-132	76,94%	KP690142.1
Gammaridae	Chaetogammarus tenellus	NA168	MH500382	Macrobrachium nipponense GTP phosphoenolpyruvate carboxykinase mRNA, partial cds	493	493	100%	3,00E-135	77,25%	KP690142.1
Niphargidae	Niphargus multipennatus	NA169	MH500383	Macrobrachium nipponense GTP phosphoenolpyruvate carboxykinase mRNA, partial cds	337	337	97%	5,00E-88	72,97%	KP690142.1
Gammaridae	Typhlogammarus mrazeki	NA175	MH500384	PREDICTED: Penaeus vannamei phosphoenolpyruvate carboxykinase, cytosolic [GTP]-like (LOC113826097), mRNA	495	495	99%	9,00E-136	77,39%	XM_027378976.1
Niphargidae	Niphargus timavi	NA190	MH500385	PREDICTED: Penaeus vannamei phosphoenolpyruvate carboxykinase, cytosolic [GTP]-like (LOC113826097), mRNA	333	333	95%	6,00E-87	72,79%	XM_027378976.1
Niphargidae	Niphargus angelieri	NA200	MH500386	PREDICTED: Penaeus vannamei phosphoenolpyruvate carboxykinase, cytosolic [GTP]-like (LOC113826097), mRNA	339	339	97%	2,00E-88	72,46%	XM_027378976.1
Niphargidae	Niphargus valachicus	NA211	MH500387	PREDICTED: Penaeus vannamei phosphoenolpyruvate carboxykinase, cytosolic [GTP]-like (LOC113819349), mRNA	351	351	100%	2,00E-92	72,80%	XM_027371589.1
Niphargidae	Niphargus pasquinii	NA221	MH500388	Macrobrachium nipponense GTP phosphoenolpyruvate carboxykinase mRNA, partial cds	330	330	95%	8,00E-86	72,53%	KP690142.1
Niphargidae	Niphargus hebereri	NA232	MH500389	PREDICTED: Penaeus vannamei phosphoenolpyruvate carboxykinase, cytosolic [GTP]-like (LOC113826097), mRNA	342	342	95%	1,00E-89	73,00%	XM_027378976.1
Niphargidae	Niphargus arbiter	NA241	MH500390	PREDICTED: Penaeus vannamei phosphoenolpyruvate carboxykinase, cytosolic [GTP]-like (LOC113826097), mRNA	347	347	95%	3,00E-91	73,07%	XM_027378976.1
Niphargidae	Niphargus balcanicus	NA246	MH500391	PREDICTED: Penaeus vannamei phosphoenolpyruvate carboxykinase, cytosolic [GTP]-like (LOC113826096), transcript variant X2, mRNA	354	354	100%	2,00E-93	73,00%	XM_027378975.1
Niphargidae	Haploginglymus sp.	NA509	MH500392	Macrobrachium nipponense GTP phosphoenolpyruvate carboxykinase mRNA, partial cds	437	437	98%	2,00E-118	75,36%	KP690142.1
Niphargidae	Niphargobates orophobata	NA546	MH500393	PREDICTED: Penaeus vannamei phosphoenolpyruvate carboxykinase, cytosolic [GTP]-like (LOC113826096), transcript variant X2, mRNA	345	345	100%	1,00E-90	72,68%	XM_027378975.1
Niphargidae	Niphargobates orophobata	NA547	MH500394	PREDICTED: Penaeus vannamei phosphoenolpyruvate carboxykinase, cytosolic [GTP]-like (LOC113826096), transcript variant X2, mRNA	345	345	100%	1,00E-90	72,68%	XM_027378975.1
Niphargidae	Niphargus stochi	NA599	MH500395	Nephrops norvegicus mRNA for Phosphoenolpyruvate carboxykinase	349	349	100%	8,00E-92	72,96%	AJ132380.1
Gammaridae	Gammarus fossarum	NA739	MH500396	Macrobrachium nipponense GTP phosphoenolpyruvate carboxykinase mRNA, partial cds	475	475	99%	8,00E-130	76,75%	KP690142.1

Niphargidae	Microniphargus leruthi	NA740	MH500397	Macrobrachium nipponense GTP phosphoenolpyruvate carboxykinase mRNA, partial cds	396	396	97%	6,00E-106	74,15%	KP690142.1
Niphargidae	Niphargus croaticus	NB013	MH500398	PREDICTED: Penaeus vannamei phosphoenolpyruvate carboxykinase, cytosolic [GTP]-like (LOC113826097), mRNA	356	356	95%	6,00E-94	73,40%	XM_027378976.1
Niphargidae	Niphargus subtypicus	NB028	MH500399	PREDICTED: Penaeus vannamei phosphoenolpyruvate carboxykinase, cytosolic [GTP]-like (LOC113826097), mRNA	356	356	95%	6,00E-94	73,40%	XM_027378976.1
Niphargidae	Niphargus steueri	NB041	MH500400	PREDICTED: Penaeus vannamei phosphoenolpyruvate carboxykinase, cytosolic [GTP]-like (LOC113826096), transcript variant X2, mRNA	350	350	97%	8,00E-92	72,79%	XM_027378975.1
Niphargidae	Microniphargus leruthi	NB090	MH500401	Macrobrachium nipponense GTP phosphoenolpyruvate carboxykinase mRNA, partial cds	396	396	97%	6,00E-106	74,15%	KP690142.1
Niphargidae	Pontoniphargus ruffoi	NB093	MH500402	Macrobrachium nipponense GTP phosphoenolpyruvate carboxykinase mRNA, partial cds	451	451	97%	3,00E-122	76,40%	KP690142.1
Niphargidae	Niphargus rhenorhodanensis	NB437	MH500403	Penaeus vannamei mRNA for phosphoenolpyruvate carboxykinase (pepck gene)	355	355	100%	2,00E-93	72,96%	AJ250829.1
Niphargidae	Niphargus brachytelson	NB621	MH500404	Nephrops norvegicus mRNA for Phosphoenolpyruvate carboxykinase	325	325	100%	1,00E-84	72,06%	AJ132380.1
Niphargidae	Niphargus podpecanus	NB910	MH500405	Nephrops norvegicus mRNA for Phosphoenolpyruvate carboxykinase	309	309	100%	7,00E-80	72,24%	AJ132380.1
Niphargidae	Niphargus glenniei	NC016	MH500406	Macrobrachium nipponense GTP phosphoenolpyruvate carboxykinase mRNA, partial cds	436	436	93%	7,00E-118	76,26%	KP690142.1
Niphargidae	Microniphargus leruthi	NC019	MH500407	Macrobrachium nipponense GTP phosphoenolpyruvate carboxykinase mRNA, partial cds	396	396	97%	6,00E-106	74,15%	KP690142.1

4. OPSIN

Family	Species	Vaucher	OPSIN - GenBank acc. number	1st BLAST hit description	Max Score	Total Score	Query cover	E value	% identity	Accession number
Crangonyctidae	Synurella ambulans	NA002	MH635367	Niphargus hrabei isolate DN16709_c0_g1_i1 putative LWS opsin mRNA, partial cds	1153	1153	100%	0	94,84%	MH521128.1
Niphargidae	Niphargus virei	NA003	MH635368	Niphargus hrabei isolate DN16709_c0_g1_i1 putative LWS opsin mRNA, partial cds	948	948	100%	0	88,97%	MH521128.1
Niphargidae	Niphargus sp.	NA006	MH635369	Niphargus hrabei isolate DN50243_c0_g1_i1 putative LWS opsin mRNA, partial cds	1139	1139	100%	0	94,17%	MH521127.1
Niphargidae	Niphargus hvarensis	NA038	MH635370	Gammarus minus isolate VS3 long-wavelength opsin (Rh) gene, partial cds	744	744	100%	0	82,36%	JX879563.1
Niphargidae	Niphargus krameri	NA039	MH635371	Niphargus hrabei isolate DN16709_c0_g1_i1 putative LWS opsin mRNA, partial cds	1175	1175	99%	0	95,38%	MH521128.1
Niphargidae	Niphargus krameri	NA040	MH635372	Niphargus hrabei isolate DN16709_c0_g1_i1 putative LWS opsin mRNA, partial cds	797	797	100%	0	95,04%	MH521128.1
Niphargidae	Niphargus rejici	NA048	MH635373	Niphargus hrabei isolate DN16709_c0_g1_i1 putative LWS opsin mRNA, partial cds	1156	1156	100%	0	93,89%	MH521128.1
Niphargidae	Niphargus zagrebensis	NA059	MH635374	Niphargus hrabei isolate DN50243_c0_g1_i1 putative LWS opsin mRNA, partial cds	1154	1154	100%	0	94,71%	MH521127.1
Niphargidae	Niphargus dalmatinus	NA060	MH635375	Niphargus hrabei isolate DN50243_c0_g1_i1 putative LWS opsin mRNA, partial cds	1127	1127	100%	0	93,89%	MH521127.1
Niphargidae	Niphargus elegans	NA061	MH635376	Niphargus hrabei isolate DN50243_c0_g1_i1 putative LWS opsin mRNA, partial cds	1119	1119	99%	0	93,73%	MH521127.1
Niphargidae	Niphargus caspary	NA073	MH635377	Niphargus hrabei isolate DN50243_c0_g1_i1 putative LWS opsin mRNA, partial cds	1141	1141	99%	0	94,42%	MH521127.1
Niphargidae	Niphargus costozzae	NA074	MH635378	Niphargus hrabei isolate DN16709_c0_g1_i1 putative LWS opsin mRNA, partial cds	1159	1159	100%	0	94,71%	MH521128.1
Niphargidae	Niphargus grandii	NA080	MH635379	Niphargus hrabei isolate DN16709_c0_g1_i1 putative LWS opsin mRNA, partial cds	847	847	99%	0	85,56%	MH521128.1
Niphargidae	Niphargus hadzii	NA082	MH635380	Niphargus hrabei isolate DN50243_c0_g1_i1 putative LWS opsin mRNA, partial cds	1154	1154	100%	0	94,71%	MH521127.1
Niphargidae	Niphargus illidzensis	NA084	MH635381	Niphargus hrabei isolate DN50243_c0_g1_i1 putative LWS opsin mRNA, partial cds	1117	1117	99%	0	93,73%	MH521127.1
Niphargidae	Niphargus subtypicus	NA112	MH635382	Niphargus hrabei isolate DN16709_c0_g1_i1 putative LWS opsin mRNA, partial cds	1153	1153	100%	0	95,06%	MH521128.1
Niphargidae	Niphargus stygius	NA123	MH635383	Niphargus hrabei isolate DN50243_c0_g1_i1 putative LWS opsin mRNA, partial cds	1164	1164	100%	0	94,84%	MH521127.1
Gammaridae	Pontogammarus abbreviatus	NA156	MH635384	Gammarus minus isolate VS1 long-wavelength opsin (Rh) gene, partial cds	847	847	100%	0	85,48%	JX879561.1
Hadziidae	Hadzia sp.	NA160	MH635385	Gammarus minus isolate HC15 long-wavelength opsin (Rh) gene, partial cds	719	719	98%	0	82,02%	JX879669.

Gammaridae	Dikerogammarus villosus	NA161	MH635386	Gammarus minus isolate DAS4 long-wavelength opsin (Rh) gene, partial cds	843	843	100%	0	85,35%	JX879532.1
Pontogammaridae	Pontogammarus crassus	NA165	MH635387	Gammarus minus isolate DAS4 long-wavelength opsin (Rh) gene, partial cds	836	836	100%	0	85,07%	JX879532.1
Pontogammaridae	Niphargoides spinicaudatus	NA167	MH635388	Gammarus minus isolate VS1 long-wavelength opsin (Rh) gene, partial cds	843	843	100%	0	85,35%	JX879561.1
Gammaridae	Chaetogammarus tenellus	NA168	MH635389	Gammarus minus isolate FRC7 long-wavelength opsin (Rh) gene, partial cds	678	678	99%	0	80,50%	JX879689.1
Niphargidae	Niphargus multipennatus	NA169	MH635390	Gammarus minus isolate VS1 long-wavelength opsin (Rh) gene, partial cds	826	826	100%	0	84,90%	JX879561.1
Gammaridae	Typhlogammarus mrazeki	NA175	MH635391	Gammarus minus isolate FRC3 long-wavelength opsin (Rh) gene, partial cds	663	663	97%	0	80,33%	JX879685.1
Niphargidae	Niphargus timavi	NA190	MH635392	Niphargus hrabei isolate DN50243_c0_g1_i1 putative LWS opsin mRNA, partial cds	1184	1184	99%	0	95,65%	MH521127.1
Niphargidae	Niphargus hebereri	NA199	MH635393	Niphargus hrabei isolate DN50243_c0_g1_i1 putative LWS opsin mRNA, partial cds	1136	1136	100%	0	94,17%	MH521127.1
Niphargidae	Niphargus pasquinii	NA221	MH635394	Niphargus hrabei isolate DN50243_c0_g1_i1 putative LWS opsin mRNA, partial cds	1150	1150	99%	0	94,69%	MH521127.1
Niphargidae	Niphargus hebereri	NA232	MH635395	Niphargus hrabei isolate DN50243_c0_g1_i1 putative LWS opsin mRNA, partial cds	1141	1141	100%	0	94,30%	MH521127.1
Niphargidae	Niphargus arbiter	NA241	MH635396	Niphargus hrabei isolate DN50243_c0_g1_i1 putative LWS opsin mRNA, partial cds	1159	1159	100%	0	94,71%	MH521127.1
Niphargidae	Niphargus stochi	NA599	MH635397	Niphargus hrabei isolate DN16709_c0_g1_i1 putative LWS opsin mRNA, partial cds	1131	1131	100%	0	94,03%	MH521128.1
Niphargidae	Carinurella paradoxa	NA738	MH635398	Niphargus hrabei isolate DN16709_c0_g1_i1 putative LWS opsin mRNA, partial cds	814	814	99%	0	84,47%	MH521128.1
Gammaridae	Gammarus fossarum	NA739	MH635399	Gammarus minus isolate VS3 long-wavelength opsin (Rh) gene, partial cds	984	984	99%	0	89,37%	JX879563.1
Niphargidae	Niphargus croaticus	NB013	MH635400	Niphargus hrabei isolate DN16709_c0_g1_i1 putative LWS opsin mRNA, partial cds	1160	1160	100%	0	94,84%	MH521128.1
Niphargidae	Niphargus subtypicus	NB028	MH635401	Niphargus hrabei isolate DN16709_c0_g1_i1 putative LWS opsin mRNA, partial cds	1165	1165	100%	0	94,98%	MH521128.1
Niphargidae	Niphargus steueri	NB041	MH635402	Niphargus hrabei isolate DN50243_c0_g1_i1 putative LWS opsin mRNA, partial cds	1186	1186	100%	0	95,66%	MH521127.1
Niphargidae	Niphargus molnari	NB554	MH635403	Niphargus hrabei isolate DN16709_c0_g1_i1 putative LWS opsin mRNA, partial cds	926	926	99%	0	87,99%	MH521128.1
Niphargidae	Niphargus brachytelson	NB621	MH635404	Niphargus hrabei isolate DN50243_c0_g1_i1 putative LWS opsin mRNA, partial cds	1133	1133	100%	0	94,26%	MH521127.1
Niphargidae	Niphargus novomestanus	NB622	MH635405	Niphargus hrabei isolate DN50243_c0_g1_i1 putative LWS opsin mRNA, partial cds	1128	1128	100%	0	94,01%	MH521127.1
Niphargidae	Niphargus podpecanus	NB910	MH635406	Niphargus hrabei isolate DN50243_c0_g1_i1 putative LWS opsin mRNA, partial cds	1139	1139	100%	0	94,53%	MH521127.1
Niphargidae	Niphargus ictus	NC026	MH635407	Niphargus hrabei isolate DN50243_c0_g1_i1 putative LWS opsin mRNA, partial cds	1150	1150	100%	0	94,57%	MH521127.1
Niphargidae	Niphargus frasassianus	NC027	MH635408	Niphargus hrabei isolate DN50243_c0_g1_i1 putative LWS opsin mRNA, partial cds	1146	1146	99%	0	94,53%	MH521127.1

5. GAPDH

Family	Species	Vaucher	GAPDH - GenBank acc. number	1st BLAST hit description	Max Score	Total Score	Query cover	E value	% identity	Accession number
Crangonyctidae	Synurella ambulans	NA002	MH668918	Alpheus armillatus voucher OUMNH.ZC.2007-20-014 glyceraldehyde 3-phosphate dehydrogenase (GAPDH) gene, partial cds	422	422	72%	1,00E-113	80,14%	MK471087.1
Niphargidae	Niphargus virei	NA003	MH668919	PREDICTED: Penaeus vannamei glyceraldehyde-3-phosphate dehydrogenase (LOC113828374), transcript variant X3, mRNA	532	532	97%	6,00E-147	79,35%	XM_027381353.1
Niphargidae	Niphargus longicaudatus	NA007	MH668920	Gammarus locusta mRNA for putative glyceraldehyde-3-phosphate dehydrogenase (gapdh gene)	725	725	100%	0	83,35%	FM165079.1
Niphargidae	Niphargus hvarensis	NA038	MH668921	Gammarus locusta mRNA for putative glyceraldehyde-3-phosphate dehydrogenase (gapdh gene)	730	730	100%	0	83,48%	FM165079.1
Niphargidae	Niphargus krameri	NA039	MH668922	Palaemon carinicauda glyceraldehyde 3-phosphate dehydrogenase mRNA, complete cds	438	438	99%	1,00E-118	76,97%	KX893516.1
Niphargidae	Niphargus krameri	NA040	MH668923	Palaemon carinicauda glyceraldehyde 3-phosphate dehydrogenase mRNA, complete cds	466	466	99%	6,00E-127	77,61%	KX893516.1
Niphargidae	Niphargus rejici	NA048	MH668924	Gammarus locusta mRNA for putative glyceraldehyde-3-phosphate dehydrogenase (gapdh gene)	713	713	100%	0	83,14%	FM165079.1
Niphargidae	Niphargus arbiter	NA052	MH668925	Gammarus locusta mRNA for putative glyceraldehyde-3-phosphate dehydrogenase (gapdh gene)	680	680	100%	0	82,36%	FM165079.1
Niphargidae	Niphargus zagrebensis	NA059	MH668926	Macrophthalmus japonicus glyceraldehyde 3-phosphate dehydrogenase mRNA, complete cds	623	623	99%	3,00E-174	81,16%	KJ653265.1
Niphargidae	Niphargus dalmatinus	NA060	MH668927	PREDICTED: Hyalella azteca glyceraldehyde-3-phosphate dehydrogenase (LOC108667228), mRNA	675	675	97%	0	82,59%	XM_018154227.1
Niphargidae	Niphargus elegans	NA061	MH668928	Gammarus locusta mRNA for putative glyceraldehyde-3-phosphate dehydrogenase (gapdh gene)	739	739	100%	0	84,07%	FM165079.1
Niphargidae	Niphargus lessiniensis	NA064	MH668929	Gammarus locusta mRNA for putative glyceraldehyde-3-phosphate dehydrogenase (gapdh gene)	702	702	100%	0	82,87%	FM165079.1
Niphargidae	Niphargus caspary	NA073	MH668930	Gammarus locusta mRNA for putative glyceraldehyde-3-phosphate dehydrogenase (gapdh gene)	725	725	100%	0	83,38%	FM165079.1
Niphargidae	Niphargus costozzae	NA074	MH668931	Gammarus locusta mRNA for putative glyceraldehyde-3-phosphate dehydrogenase (gapdh gene)	702	702	100%	0	82,85%	FM165079.1
Niphargidae	Niphargus grandii	NA080	MH668932	PREDICTED: Hyalella azteca glyceraldehyde-3-phosphate dehydrogenase (LOC108667228), mRNA	702	702	97%	0	83,27%	XM_018154227.1
Niphargidae	Niphargus kochianus	NA090	MH668933	Gammarus locusta mRNA for putative glyceraldehyde-3-phosphate dehydrogenase (gapdh gene)	824	824	100%	0	85,66%	FM165079.1
Niphargidae	Niphargus orcinus	NA099	MH668934	Gammarus locusta mRNA for putative glyceraldehyde-3-phosphate dehydrogenase (gapdh gene)	693	693	100%	0	83,07%	FM165079.1
Niphargidae	Niphargus spinulifemur	NA107	MH668935	Gammarus locusta mRNA for putative glyceraldehyde-3-phosphate dehydrogenase (gapdh gene)	691	691	100%	0	82,61%	FM165079.1
Niphargidae	Niphargus vjetrenicensis	NA116	MH668936	Gammarus locusta mRNA for putative glyceraldehyde-3-phosphate dehydrogenase (gapdh gene)	691	691	100%	0	82,99%	FM165079.1

Niphargidae	Niphargus stygius	NA123	MH668937	Gammarus locusta mRNA for putative glyceraldehyde-3-phosphate dehydrogenase (gapdh gene)	756	756	99%	0	84,18%	FM165079.1
Gammaridae	Dikerogammarus villosus	NA161	MH668938	Gammarus locusta mRNA for putative glyceraldehyde-3-phosphate dehydrogenase (gapdh gene)	915	915	99%	0	87,77%	FM165079.1
Pontogammaridae	Pontogammarus crassus	NA165	MH668939	Gammarus locusta mRNA for putative glyceraldehyde-3-phosphate dehydrogenase (gapdh gene)	904	904	100%	0	87,31%	FM165079.1
Pontogammaridae	Niphargoides spinicaudatus	NA167	MH668940	Gammarus locusta mRNA for putative glyceraldehyde-3-phosphate dehydrogenase (gapdh gene)	889	889	100%	0	87,07%	FM165079.1
Gammaridae	Chaetogammarus tenellus	NA168	MH668941	Gammarus locusta mRNA for putative glyceraldehyde-3-phosphate dehydrogenase (gapdh gene)	937	937	100%	0	88,06%	FM165079.1
Gammaridae	Typhlogammarus mrazeki	NA175	MH668942	Gammarus locusta mRNA for putative glyceraldehyde-3-phosphate dehydrogenase (gapdh gene)	1026	1026	99%	0	90,20%	FM165079.1
Niphargidae	Niphargus timavi	NA190	MH668943	Litopenaeus vannamei glyceraldehyde-3-phosphate dehydrogenase (GAPDH) gene, partial cds	538	538	97%	1,00E-148	79,61%	MG878889.1
Niphargidae	Niphargus hebereri	NA199	MH668944	PREDICTED: Penaeus vannamei glyceraldehyde-3-phosphate dehydrogenase (LOC113828374), transcript variant X3, mRNA	514	514	97%	2,00E-141	79,04%	XM_027381353.1
Niphargidae	Niphargus glenniei	NA208	MH668945	PREDICTED: Penaeus vannamei glyceraldehyde-3-phosphate dehydrogenase (LOC113828374), transcript variant X3, mRNA	662	662	100%	0	82,03%	XM_027381353.1
Niphargidae	Niphargus pasquinii	NA221	MH668946	Gammarus locusta mRNA for putative glyceraldehyde-3-phosphate dehydrogenase (gapdh gene)	701	701	100%	0	82,72%	FM165079.1
Niphargidae	Niphargus hebereri	NA232	MH668947	PREDICTED: Penaeus vannamei glyceraldehyde-3-phosphate dehydrogenase (LOC113828374), transcript variant X3, mRNA	499	499	97%	6,00E-137	78,67%	XM_027381353.1
Niphargidae	Niphargus balcanicus	NA246	MH668948	Gammarus locusta mRNA for putative glyceraldehyde-3-phosphate dehydrogenase (gapdh gene)	723	723	100%	0	83,42%	FM165079.1
Niphargidae	Haploginglymus sp.	NA509	MH668949	Nautilocaris saintlaurentae glyceraldehyde 3-phosphate dehydrogenase mRNA, partial cds	407	407	91%	4,00E-109	77,21%	MH663451.1
Niphargidae	Niphargus glenniei	NA524	MH668950	PREDICTED: Penaeus vannamei glyceraldehyde-3-phosphate dehydrogenase (LOC113828374), transcript variant X3, mRNA	662	662	100%	0	82,03%	XM_027381353.
Niphargidae	Niphargus fongi	NA538	MH668951	Gammarus locusta mRNA for putative glyceraldehyde-3-phosphate dehydrogenase (gapdh gene)	795	795	99%	0	85,06%	FM165079.1
Niphargidae	Haploginglymus sp.	NA543	MH668952	Nautilocaris saintlaurentae glyceraldehyde 3-phosphate dehydrogenase mRNA, partial cds	407	407	91%	4,00E-109	77,21%	MH663451.1
Niphargidae	Niphargobates orophobata	NA546	MH668953	Gammarus locusta mRNA for putative glyceraldehyde-3-phosphate dehydrogenase (gapdh gene)	601	601	100%	2,00E-167	80,58%	FM165079.1
Niphargidae	Niphargobates orophobata	NA547	MH668954	Gammarus locusta mRNA for putative glyceraldehyde-3-phosphate dehydrogenase (gapdh gene)	617	617	100%	1,00E-172	80,96%	FM165079.1
Niphargidae	Niphargus stochi	NA599	MH668955	Galathea squamifera voucher OUMNH:2002-17-0006 glyceraldehyde 3-phosphate dehydrogenase (GAPDH) gene, partial cds	446	446	82%	7,00E-121	79,10%	MG596247.1
Niphargidae	Carinurella paradoxa	NA738	MH668956	Gammarus locusta mRNA for putative glyceraldehyde-3-phosphate dehydrogenase (gapdh gene)	730	730	99%	0	83,55%	FM165079.1
Gammaridae	Gammarus fossarum	NA739	MH668957	Gammarus pulex glyceraldehyde 3-phosphate dehydrogenase (GAPDH) partial sequence	1136	1136	82%	0	98,30%	DQ272519.2
Niphargidae	Microniphargus leruthi	NA740	MH668958	Gammarus pulex glyceraldehyde 3-phosphate dehydrogenase (GAPDH) partial sequence	442	442	81%	1,00E-119	78,73%	DQ272519.2
Niphargidae	Microniphargus leruthi	NA741	MH668959	Gammarus pulex glyceraldehyde 3-phosphate dehydrogenase (GAPDH) partial sequence	453	453	81%	4,00E-123	79,56%	DQ272519.2

Crangonycidae	Synurella sp.	NB091	MH668960	Palaemon carinicauda glyceraldehyde 3-phosphate dehydrogenase mRNA, complete cds	547	547	89%	2,00E-151	80,76%	KX893516.1
Niphargidae	Pontoniphargus ruffoi	NB093	MH668961	PREDICTED: Hyalella azteca glyceraldehyde-3-phosphate dehydrogenase (LOC108667228), mRNA	675	675	97%	0	82,61%	XM_018154227.1
Niphargidae	Pontoniphargus racovitzai	NB094	MH668962	PREDICTED: Hyalella azteca glyceraldehyde-3-phosphate dehydrogenase (LOC108667228), mRNA	665	665	99%	0	81,95%	XM_018154227.1
Niphargidae	Niphargellus nolli	NB365	MH668963	Nautilocaris saintlaurentae glyceraldehyde 3-phosphate dehydrogenase mRNA, partial cds	401	401	91%	2,00E-107	77,07%	MH663451.1
